# Multiomics characterization implicates PTK7 in ovarian cancer EMT and cell plasticity and offers strategies for therapeutic intervention

**DOI:** 10.1038/s41419-022-05161-5

**Published:** 2022-08-17

**Authors:** Juuli Raivola, Alice Dini, Hanna Karvonen, Emilia Piki, Kari Salokas, Wilhelmiina Niininen, Laura Kaleva, Kaiyang Zhang, Mariliina Arjama, Greta Gudoityte, Brinton Seashore-Ludlow, Markku Varjosalo, Olli Kallioniemi, Sampsa Hautaniemi, Astrid Murumägi, Daniela Ungureanu

**Affiliations:** 1 Applied Tumor Genomics, Research Program Unit, Faculty of Medicine, University of Helsinki, 00014 Helsinki, Finland; 2 Cancer Signaling, Faculty of Medicine and Health Technology, Tampere University, 33014 Tampere, Finland; 3 Institute of Biotechnology, University of Helsinki, 00014 Helsinki, Finland; 4 Research Program in Systems Oncology, Research Program Unit, Faculty of Medicine, University of Helsinki, 00014 Helsinki, Finland; 5 Institute for Molecular Medicine, FIMM, Helsinki Institute of Life Science (HiLIFE), University of Helsinki, 00014 Helsinki, Finland; 6 Science for Life Laboratory, Department of Oncology and Pathology, Karolinska Institutet, 17121 Stockholm, Sweden; 7 Faculty of Biochemistry and Molecular Medicine, University of Oulu, 90014 Oulu, Finland

**Keywords:** Cancer, Cell signalling

## Abstract

Most patients with ovarian cancer (OC) are diagnosed at a late stage when there are very few therapeutic options and a poor prognosis. This is due to the lack of clearly defined underlying mechanisms or an oncogenic addiction that can be targeted pharmacologically, unlike other types of cancer. Here, we identified protein tyrosine kinase 7 (PTK7) as a potential new therapeutic target in OC following a multiomics approach using genetic and pharmacological interventions. We performed proteomics analyses upon PTK7 knockdown in OC cells and identified novel downstream effectors such as synuclein-γ (SNCG), SALL2, and PP1γ, and these findings were corroborated in ex vivo primary samples using PTK7 monoclonal antibody cofetuzumab. Our phosphoproteomics analyses demonstrated that PTK7 modulates cell adhesion and Rho-GTPase signaling to sustain epithelial-mesenchymal transition (EMT) and cell plasticity, which was confirmed by high-content image analysis of 3D models. Furthermore, using high-throughput drug sensitivity testing (525 drugs) we show that targeting PTK7 exhibited synergistic activity with chemotherapeutic agent paclitaxel, CHK1/2 inhibitor prexasertib, and PLK1 inhibitor GSK461364, among others, in OC cells and ex vivo primary samples. Taken together, our study provides unique insight into the function of PTK7, which helps to define its role in mediating aberrant Wnt signaling in ovarian cancer.

## Introduction

The reactivation of developmental pathways such as Wnt signaling that are critical for embryogenesis and fetal development is an important feature of cancer. The contribution of Wnt pathway to tumorigenesis and drug resistance has been well documented in numerous cancers [1]. Among the Wnt receptors, there are members of the receptor tyrosine kinase (RTK) family such as receptor tyrosine kinase-like orphan receptor 1 and 2 (ROR1 and 2), protein tyrosine kinase 7 (PTK7), and receptor-like tyrosine kinase (RYK) [2], which are also considered as pseudokinases owing to substitutions of conserved and essential catalytic residues required for phosphotransferase activity [3]. These catalytically impaired receptors are dysregulated in multiple diseases, and changes in expression levels of ROR1, ROR2, PTK7, and RYK are often associated with malignant transformation, suggesting a great therapeutic utility for these molecules [4]. However, therapeutic development has been limited by a lack of mechanistic understanding of these receptors signaling. Our previous studies have shown that the pseudokinase domains of ROR1, ROR2, PTK7, and RYK are structurally and dynamically related to their nearest kinase-active homolog, the insulin receptor, displaying similar conformational plasticity [2].

Aberrant activation of non-canonical Wnt signaling has been detected in ovarian cancer (OC) [5]. More specifically, numerous studies have demonstrated that Wnt5a binding to ROR1 and ROR2 promotes OC tumorigenesis and therapy resistance [6–9]. However, investigations into the possible functional and therapeutic role of PTK7 in OC have not benefited from the same attention as for ROR1/2 receptors. Earlier studies pointed towards a tumor suppressor role for PTK7 in OC [10], while recent clinical data from PTK7 targeting with the antibody-drug conjugate cofetuzumab-pelidotin showed promising outcomes in phase I clinical trial for solid tumors, including chemoresistant OC patients [11]. Notably, given that PTK7 binds the same Wnt5a ligand as ROR1 and ROR2, it is conceivable that PTK7 may also be involved in OC progression and therapeutic modulation. Moreover, overexpression of PTK7 has been found in colon, lung, and gastric cancers that are known to display Wnt signaling aberrations [12], which indicates that this receptor is clearly involved in tumorigenic mechanisms and warrants careful investigation.

OC is the second most lethal gynecological cancer in developed countries and comprises of type I and type II tumors, each with distinct molecular profile and treatment outcome [13]. Type I OC tumors encompass the subtypes low-grade serous ovarian cancer (LGSC), endometrioid, mucinous, and clear cell. This type is characterized by high genomic stability, a p53 wild-type expression, and has mutations in various pathways including genes such as *KRAS, BRAF, PTEN, PIK3CA*, and *ARID1A* [14]. Type II OC tumors include high-grade serous ovarian cancer (HGSC, accounts for around 75% of OC cases), high-grade endometrioid, undifferentiated cancers, and carcinosarcomas, which are defined by high genomic instability, a nearly 100% p53 mutation rate, defects in homologous recombination (HR) repair, mutations in *BRCA1/2*, and extensive copy number aberrations [15]. The current standard of care for patients with OC involves debulking surgery and platinum-based chemotherapy usually in combination with a taxane [16]. While type II OC tumors are initially more responsive to platinum-based chemotherapy, they tend to recur and become resistant to chemotherapy, and are responsible for the majority of deaths from OC [15].

In this study, we provide insight into the role of PTK7 in OC tumorigenesis and compare its oncogenic potential to that of ROR1 and ROR2, the other two Wnt5a-binding receptor pseudokinases. We applied a multiomics approach using genetic knockdown or treatment with PTK7 monoclonal antibody (mAb) cofetuzumab to target its expression in OC cell lines and patient-derived cell cultures (PDCs) ex vivo from both type I and type II OC tumors. We identified downstream signaling specificities for PTK7 and validated its functional role in regulating cell migration, EMT, and cell plasticity. In addition, we found drug combinations via (co-)targeting PTK7 that could overcome OC treatment resistance. Our study describes how PTK7 expression provides a niche for cancer cells to sustain tumorigenesis, and highlights this molecule as a candidate target to improve the therapeutic outcome in OC.

## Materials and methods

### Ethics approval and consent to participate

All patient material and data were acquired under Institutional Ethical Review Board-approved protocols (No. 56/13/03/03/2014). The study and the use of all clinical material have been approved by The Ethics Committee of the Hospital District of Southwest Finland (ETMK) under decision number ETMK: 145/1801/2015.

### Patient derived cell cultures (PDCs)

All patients’ clinical characteristics are provided in Supplementary Fig. 1B. PDCs were cultured as previously described [17] and verified for identical phenocopy with their original tumor samples by next-generation sequencing (data not shown) and the expression of Müllerian marker paired-box 8 (PAX8), a known marker for the diagnosis of epithelial ovarian cancers.

More information on materials and methods is available in Supplementary Materials and Methods, and whole blots for the immunoblots shown in the main figures are shown in Supplementary Fig. 7.

## Results

### PTK7 expression is detected in relapsed OC primary tumors and correlates with poor prognosis

**Fig. 1:**
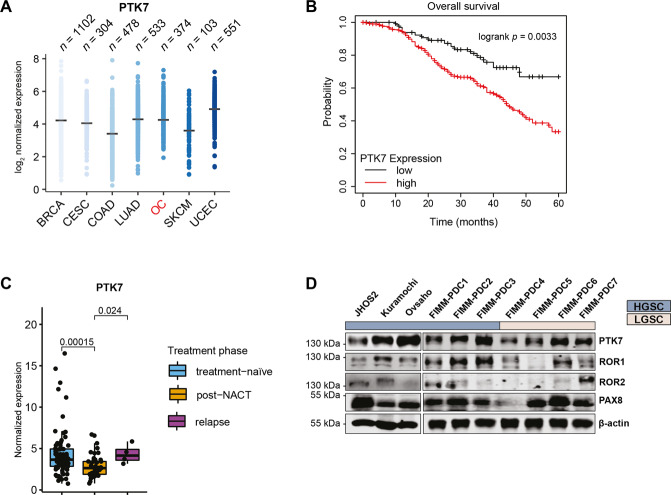
The expression and clinical significance of PTK7 in OC. **A** Expression profile of PTK7 in the seven most common cancers in women from The Cancer Genome Atlas (TCGA): BRCA (breast invasive carcinoma), COAD (colon adenocarcinoma), LUAD (lung adenocarcinoma), OC (ovarian serous cystadenocarcinoma), SKCM (skin cutaneous melanoma), and UCEC (uterine corpus endometrial carcinoma). **B** Kaplan-Meier analysis [53] of GSE9891 dataset shows that PTK7 expression in OC tumors is significantly correlated with poor overall survival. **C** Expression levels of PTK7 in treatment-naïve (*n* = 75), post-NACT (*n* = 46), and relapsed (*n* = 4) HGSC patient samples in the DECIDER cohort; *p*-values were calculated using the Wilcoxon rank sum test. **D** Immunoblot analysis of ROR1, ROR2, and PTK7 expression in HGSC and LGSC cell lines and PDCs. β-actin was used as loading control. A representative of 3 independent immunoblots is shown.

Pan-cancer TCGA analysis of PTK7 expression in the seven most common cancers in women (breast, cervical, colon, lung, ovarian, skin, and uterine cancers) revealed that PTK7 is highly expressed in all selected cancers (Fig. 1A). PTK7 transcriptomic levels were also increased in primary tumors compared to normal tissues of these commonly occurring cancers (Supplementary Fig. 1A). High PTK7 expression in OC correlated with lower overall survival (OS) (logrank *p* = 0.0033) by Kaplan-Meier analysis of GSE9891 dataset (*n* = 285) [18] consisting of HGSC and LGSC samples (Fig. 1B). Transcriptomic analysis of the DECIDER cohort identified lower PTK7 levels (*p* = 0.00015) in post-NACT (*n* = 46) compared to treatment-naïve (*n* = 75) tumors (Fig. 1C), however, its expression was elevated again in relapsed OC samples (*p* = 0.024).

Furthermore, the expression of PTK7 and the other two related non-canonical Wnt5a signaling receptors, ROR1 and ROR2, was assessed by immunoblotting in three HGSC representative cell lines (JHOS2, Kuramochi, and Ovsaho), and seven patient-derived cell cultures (PDCs) comprising of both type I (LGSC) and type II (HGSC) tumors (Fig. 1D, Supplementary Fig. 1B). PTK7 expression was detected at variable levels in all PDCs, whereas ROR1 and ROR2 were less prevalent, suggesting that this receptor is present in both HGSC and LGSC tumors. All PDCs showed the expression of OC marker PAX8 (Fig. 1D).

### Proteomics analyses identified downstream effectors of PTK7 in OC cells

**Fig. 2:**
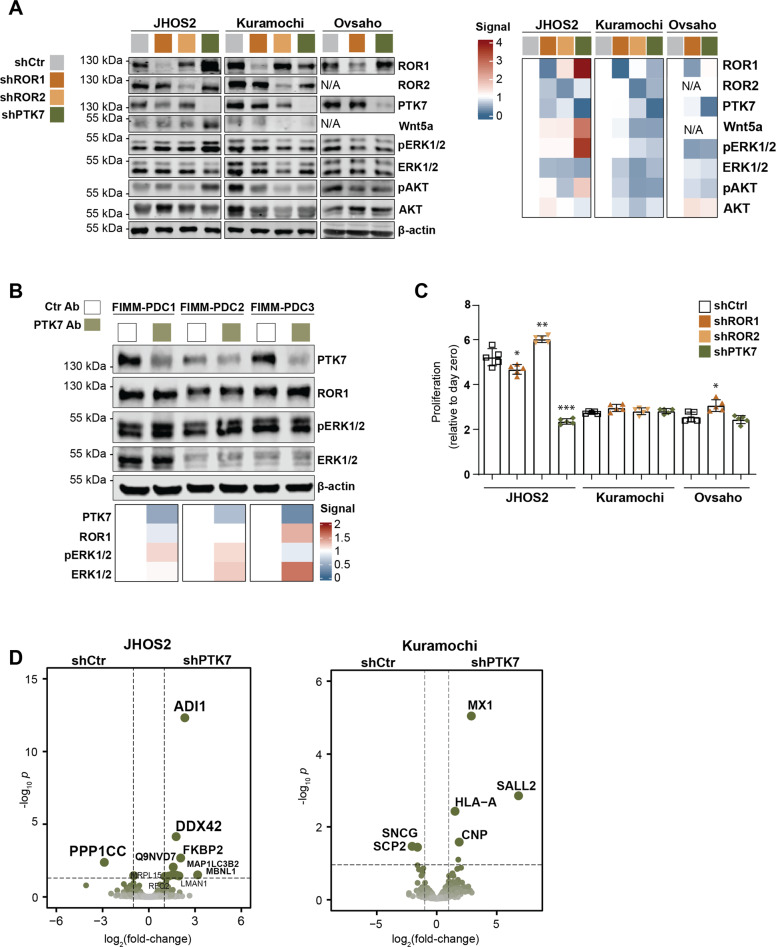
Proteomics data analysis identifies downstream effectors of PTK7 in OC. **A** Immunoblot analysis of DOX-treated (4 days) JHOS2, Kuramochi, and Ovsaho shRNA cell lysates. Protein levels were normalized first based on β-actin, which was used as loading control, and then to shCtr samples for each cell line (set as 1). A representative of 3 independent experiments is shown. **B** Immunoblot analysis of FIMM-PDC1-3 cell lysates treated with control or PTK7 mAb cofetuzumab (10 µg/ml) for 48 h. For signal quantification, protein levels were normalized based on β-actin that was used as loading control. **C** Cell viability assay of DOX-treated (7 days) shRNA cell lines. Graphs show mean (*n* = 4 biological replicates) ± SD; *p-*values (two-tailed Student´s *t*-test): *p ≤ 0.05, **p ≤ 0.01, ***p ≤ 0.001. **D** Volcano plots showing the distribution of the protein level fold-changes and *p*-values for the comparisons of differentially expressed proteins (DEPs) in shPTK7 vs. shCtr samples for each cell line; *p* < 0.05.

To identify downstream signaling specificities mediated by PTK7 in OC cells, we used JHOS2, Kuramochi, and Ovsaho cell lines and performed doxycycline (DOX)-inducible stable shRNA-mediated knockdown of PTK7, ROR1, or ROR2. This approach allowed us to assess PTK7 signaling in a more comprehensive way and side-by-side with other non-canonical Wnt5a receptors. Interestingly, we noticed a strong upregulation of ROR1 and its associated Wnt5a and pERK/pAKT signaling in JHOS2 shPTK7 cell lysates (Fig. 2A), and this effect was confirmed using two different shRNAs (Supplementary Fig. 2A) as well as by flow-cytometry analysis of JHOS2 shPTK7 cells (Supplementary Fig. 2B). To investigate whether the same effect is observed by targeting PTK7 with monoclonal antibody (mAb), we used cofetuzumab that efficiently binds to and induces PTK7 downregulation [19]. Cofetuzumab treatment upregulated pERK/pAKT but not ROR1 levels in JHOS2 (Supplementary Fig. 2C), whereas upregulation of ROR1 was observed in FIMM-PDC3, which expressed high PTK7 levels (Fig. 2B). Notably, targeting ROR1 did not result in significant PTK7 expression alteration (Fig. 2A).

Next, we addressed the functional relevance of PTK7 knockdown and assessed the long-term survival of JHOS2, Kuramochi, and Ovsaho stably-transduced shRNA cell lines after 7 days of DOX treatment. Strikingly, a marked loss of cell proliferation was observed in JHOS2 shPTK7 cells (50% reduction of cell proliferation as compared to day 0, *p* = 0.001), whereas knockdown of either ROR1 or ROR2 had no effect on cell survival in any of the tested shRNA cell lines, and a similar outcome was obtained for Kuramochi and Ovsaho shPTK7 cells (Fig. 2C).

To uncover the molecular mechanisms mediated by PTK7 in OC cells, we performed LC-MS/MS-based proteomics analyses of JHOS2 and Kuramochi shPTK7 cell lines. Comparison of differentially expressed proteins (DEPs, log_2_ fold-change, *p* < 0.05) of shPTK7 samples with shCtr samples of the same cell line identified several molecular effectors (Fig. 2D). For example, downregulation of PPP1CC/PP1γ, the γ-subunit of the serine/threonine-protein phosphatase PP1 that plays an essential role in cell division and protein turnover via dephosphorylation [20], and upregulation of ADI1 (1,2-dihydroxy-3-keto-5-methylthiopentene dioxygenase), a protein involved in L-methionine synthesis previously shown to act as tumor suppressor in prostate cancer [21] were observed in JHOS2 shPTK7 cells. Furthermore, downregulation of synuclein-γ (SNCG), a G-protein coupled receptor (GPCR) substrate associated with advanced disease and chemoresistance in multiple solid tumors, including OC [22] was observed in Kuramochi shPTK7 cells (Fig. 2D). We also noticed upregulation of SALL2 following PTK7 knockdown in Kuramochi cells. SALL2 is a member of the SALL transcription factor family with a tumor suppressor role in ovarian cancer cells [23].

### SNCG and PP1γ are downstream targets of PTK7 in OC

**Fig. 3:**
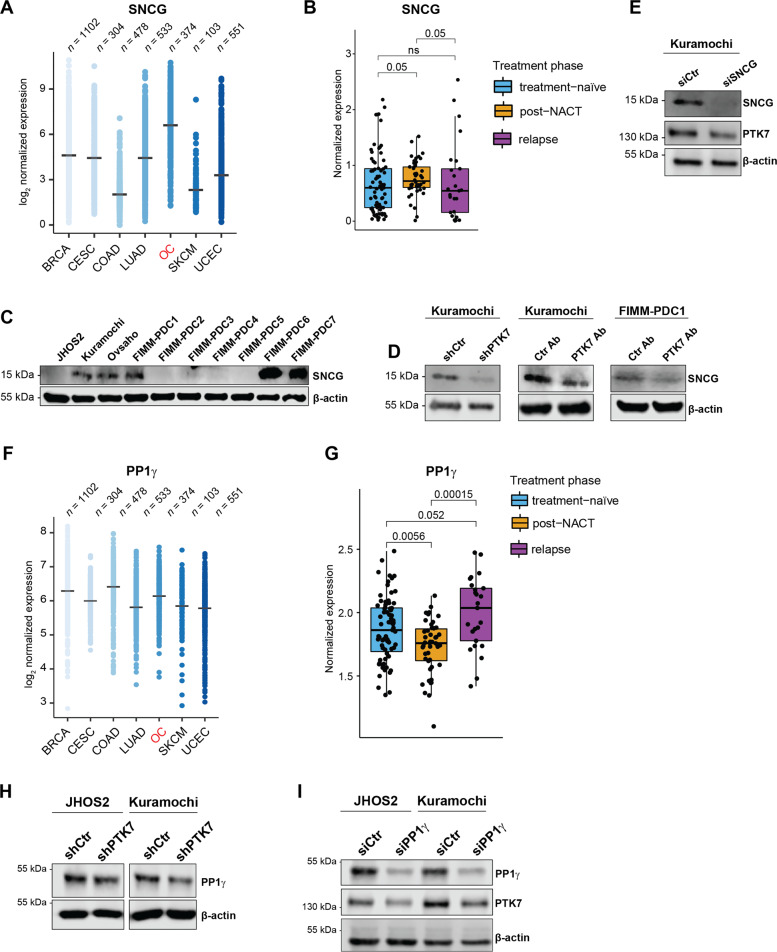
SNCG and PP1γ are downstream targets for PTK7 and linked to disease progression OC. **A** TCGA expression profile of SNCG in the seven most common cancers in women (*n* = number of samples): BRCA (breast invasive carcinoma), COAD (colon adenocarcinoma), LUAD (lung adenocarcinoma), OC (ovarian serous cystadenocarcinoma), SKCM (skin cutaneous melanoma), and UCEC (uterine corpus endometrial carcinoma). **B** Expression levels of SNCG in treatment-naïve (*n* = 75), post-NACT (*n* = 46), and relapsed (*n* = 4) HGSC patient samples in the DECIDER cohort; *p*-values were calculated using the Wilcoxon rank sum test. **C** Immunoblot analysis of SNCG expression in HGSC and LGSC cell lines and PDCs. **D** Immunoblot analysis of Kuramochi shRNA samples and Kuramochi and FIMM-PDC1 treated for 48 h with 10 µg/ml of Ctr mAb or PTK7 mAb cofetuzumab. **E** Immunoblot analysis of Kuramochi cell lysates following siRNA Ctr or SNCG transfection for 72 h. **F** TCGA expression profile of PP1γ in the seven most common cancers in women (*n* = number of samples, abbreviations as in **A**). **G** Expression levels of PP1γ in treatment-naive (*n* = 75), post-NACT (*n* = 46), and relapsed (*n* = 4) HGSC patient samples in DECIDER cohort; *p*-values were calculated using the Wilcoxon rank sum test. **H** Immunoblot analysis of PP1γ levels in JHOS2 and Kuramochi shCtr and shPTK7 cell lines induced with DOX for 4 days. **I** Immunoblot analysis of the OC cell lines transiently transfected for 72 h with siRNA Ctr or PP1γ. For **C**–**E** and **H, I** β-actin was used as loading control, and a representative of 3 independent immunoblots is shown.

Our proteomics analyses identified SNCG and PP1γ as downregulated targets following PTK7 knockdown in Kuramochi and JHOS2 cells, respectively. Previous reports have indicated that high levels of SNCG significantly correlated with worse progression-free survival (PFS) and overall survival (OS) in HGSC patients by activating PI3K/AKT pathway [22], and the same effect was observed in breast and cervical cancers [24, 25]. In the TCGA cohort, we identified higher SNCG expression in OC compared to the other six most common cancers in women (Fig. 3A) and increased SNCG levels were found in OC tumors versus normal fallopian tube tissues where HGSC is thought to originate [26, 27] (Supplementary Fig. 3A). In addition, significantly higher levels of SNCG were found in post-NACT versus treatment-naïve patient samples in the DECIDER cohort (*p* = 0.05), corroborating previous findings associating SNCG expression with OC chemoresistance (Fig. 3B) [22]. Interestingly, immunoblot analysis showed variable SNCG protein levels in OC cell lines and PDCs (Fig. 3C). In line with our proteomics data, downregulation of SNCG was observed in Kuramochi shPTK7 cell line, as well as in Kuramochi and FIMM-PDC1 cells upon PTK7 targeting with mAb cofetuzumab (Fig. 3D). On the other hand, siRNA-mediated SNCG targeting in Kuramochi cells resulted in PTK7 downregulation (Fig. 3E), which suggests the existence of a regulatory feedback loop between PTK7 and SNCG.

Moreover, our proteomics data identified the downregulation of PP1γ in JHOS2 shPTK7 cells. High expression of PP1γ was detected in all seven most common cancers in women (Fig. 3F), and elevated PP1γ levels were prevalently found in OC tumors versus normal fallopian tube tissues (Supplementary Fig. 3A). Similarly to PTK7, PP1γ expression observed in the DECIDER cohort showed decreased levels in post-NACT but increased levels in relapsed compared to treatment-naïve samples (Fig. 3G). Corroborating our proteomics data, decreased PP1γ levels were observed in JHOS2 as well as Kuramochi shPTK7 compared to the corresponding shCtr samples (Fig. 3H). Functionally relevant, siRNA-mediated downregulation of PP1γ resulted in PTK7 downregulation in both JHOS2 and Kuramochi cells (Fig. 3I).

Next, we sought to investigate whether PTK7 and its downstream SNCG and PP1γ effectors are modulated by Wnt5a, which is known to mediate non-canonical Wnt signaling in multiple tumors, including OC [28, 29]. For this purpose, serum-starved and quiescent OC cells (JHOS2, Kuramochi, FIMM-PDC1, and FIMM-PDC3) were treated with recombinant Wnt5a (100 ng/ml) for various time points, and the levels of PTK7, SNCG, and PP1γ were assessed via immunoblotting (Supplementary Fig. 3B). Our results show a Wnt5a-mediated increase in PTK7 levels in all OC models, and the same effect, although more variable, was observed for SNCG and PP1γ levels. Taken together, our data show that PTK7 signaling is transcriptionally upregulated by its Wnt5a ligand in OC cells.

### Phosphoproteomics reveals a role for non-canonical Wnt5a signaling in EMT and Rho-GTPases modulation

**Fig. 4:**
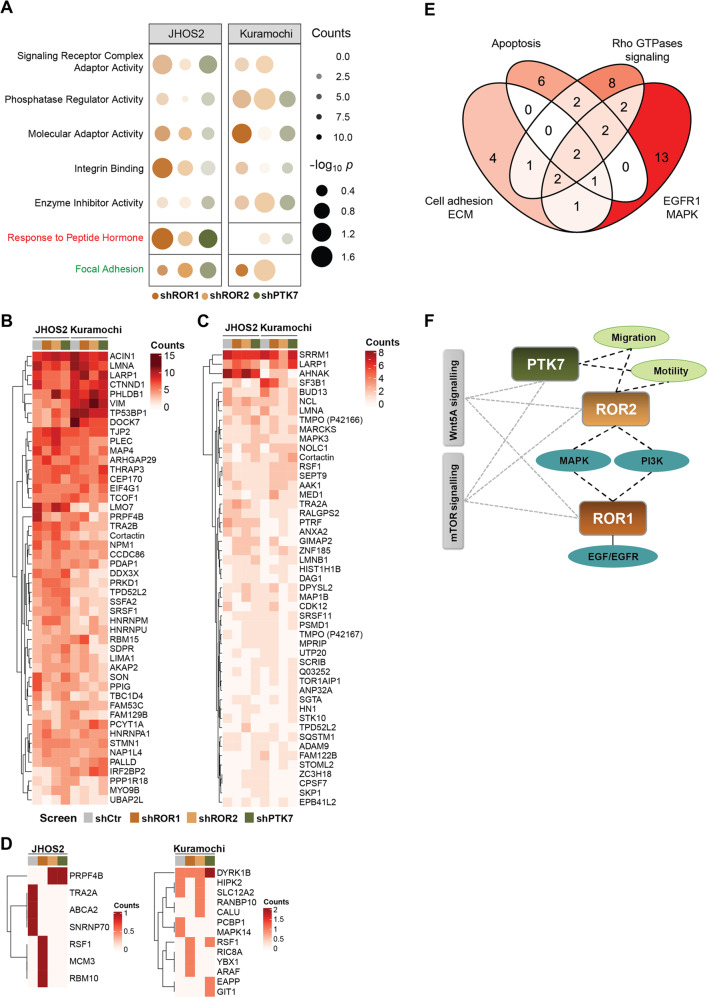
Phosphoproteomics uncovers specific signaling dynamics associated with PTK7, ROR1, and ROR2 expression in OC cells. **A** Commonly identified molecular functions (black), biological processes (red) and cellular components (green) following phosphoproteomics and functional enrichment analysis of shROR1, shROR2, and shPTK7 samples compared to shCtr samples in both JHOS2 and Kuramochi cell lines. **B**–**D** Heatmaps of dysregulated phosphoproteins in terms of phospho-serine (**B**), -threonine (**C**), and -tyrosine (**D**) in shRNA JHOS2 and Kuramochi cell lines. For a better visualization, AHNAK and TNKS1PB1 were left out from the heatmap **B**, whereas SRRM2 was left out from the heatmap **C**, but they were included in all analyses. **E** Venn diagram depicting the top four dysregulated pathways among the phosphoproteins identified in **B**–**D**. **F** Schematic representation of the terms associated with PTK7, ROR1, and ROR2, based on GO functional enrichment analysis. The terms specific to one receptor are linked by solid lines, whereas terms that are unique to a receptor pair are connected by dashed lines.

Next, we studied the phosphoproteome regulated by PTK7, ROR1, and ROR2 in JHOS2 and Kuramochi cells as to obtain insight into the non-canonical Wnt5a signaling in OC. We focused on phosphopeptides displaying differences in their counts and identified functional mechanisms associated with signaling receptor complex adaptors, molecular adaptor, phosphatase regulator activity, enzyme inhibitor activity, integrin binding, and focal adhesion (Fig. 4A). Moreover, for the upregulated and downregulated phospho-counts, the distribution of the three phosphorylated residues (serine, threonine, and tyrosine) showed more up-regulated phospho-tyrosine counts in shPTK7 Kuramochi and shROR1 JHOS2 samples compared to other shRNA samples from the same cell line (Supplementary Fig. 4A).

We then analyzed the top 50 dysregulated phosphoproteins with highest abundance of phospho-serine and phospho-threonine counts, whereas for phospho-tyrosine counts all hits were included (Fig. 4B–D, Supplementary Fig. 4B). Pathway-oriented over-representation analysis highlighted four pathways: EGFR1/MAPK signaling, cell adhesion and EMT, Rho-GTPases signaling, and apoptosis (Fig. 4E). Furthermore, specific terms related to PTK7 and/or ROR1, ROR2, and Wnt5a (Fig. 4E, Supplementary Table 1) showed that PTK7 enriched with terms related to cell motility, adhesion, and migration. Terms related to MAPK, PI3K, and EGF/EGFR signaling were distinctively enriched with ROR1 and ROR2, but not with PTK7, whereas all three receptors were linked to Wnt5a and mTOR signaling modules (Fig. 4F).

### Targeting PTK7 impairs migration and EMT processes in OC

**Fig. 5:**
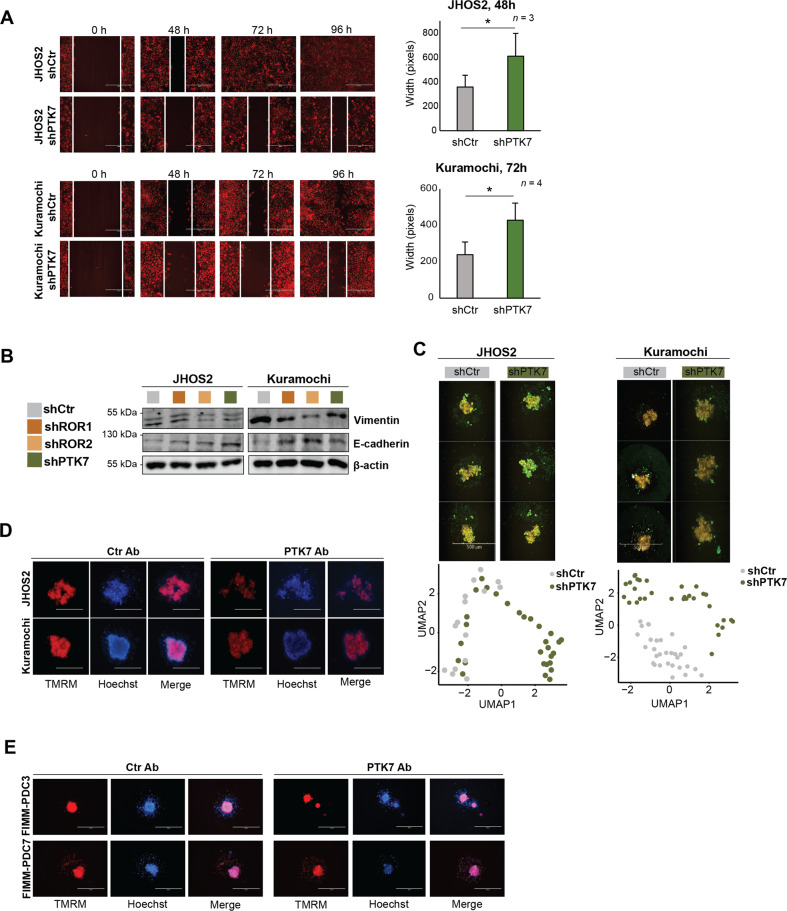
Targeting PTK7 impairs migration, EMT, and spheroid formation. **A** Wound-healing assay of JHOS2 and Kuramochi shCtr and shPTK7 cells induced with 100 ng/ml DOX for 6 days prior the addition of TMRE stain. After 24 h, a wound was created, and the healing was observed every 24 h. Scale bar: 400 µm. Right, analysis of the width of the wound was performed with ImageJ and the graphs show mean (*n* = images from biological duplicates) ± SD; *p-*values (two-tailed Student´s *t*-test): *p ≤ 0.05 **B** Immunoblot analysis of shRNA JHOS2 and Kuramochi cell lines showing the expression of vimentin and E-cadherin. β-actin was used as loading control. **C** Representative immunofluorescence images of TMRM (yellow) and Dioc6(3) (green) staining (upper panels) and UMAP of high-content image data analysis (lower panels) of JHOS2 and Kuramochi shCtr and shPTK7 spheroids. Scale bar: 500 µm. **D**, **E**. Representative immunofluorescence images of TMRM (red) and Hoechst (blue, nuclear staining) of OC cell lines (**D**) or PDCs **E** treated with 20 µg/ml control mAb or PTK7 mAb (cofetuzumab) for 5 days. Scale bar: 500 µm.

Since we identified that EMT, cell adhesion, and motility are the top functional processes mediated by non-canonical Wnt receptors, we sought to determine more in detail this phenotypic regulation. First, we investigated whether targeting PTK7 affects cell migration by performing wound-healing assay. Our results clearly showed that the wound closure was significantly affected in shPTK7 JHOS2 and Kuramochi cells compared with shCtr cells (Fig. 5A). Immunoblot analysis revealed an induction of E-cadherin and loss of vimentin following PTK7, ROR1, and ROR2 knockdown, corresponding to an epithelial phenotype and a loss of mesenchymal properties (Fig. 5B), suggesting a strong modulation of EMT. Next, we cultured JHOS2 and Kuramochi shRNA cells in low-attachment plates to examine spheroid formation upon receptor targeting. Phenotypic analysis of JHOS2 and Kuramochi shRNA spheroids revealed morphological differences such as spheroids with more scattered edges and disseminated structure following PTK7 knockdown compared to shCtr samples (Supplementary Fig. 5A). These effects were further illustrated by UMAP representation using high-content image data analysis (HCiA) showing distinct phenotypic clusters of shCtr and shPTK7 spheroids assessed both in JHOS2 and Kuramochi cell lines (Fig. 5C). The disruptive effect of receptor targeting on spheroid formation was even more pronounced in JHOS2 shRNA spheroids grown for long time (weeks) in solid matrix such as matrigel (Supplementary Fig. 5B). Our results showed statistically significant morphologic differences in matrigel-grown JHOS2 spheroids where shPTK7 cells failed to form proper spheroids, whereas shROR1 and shROR2 cells formed smaller spheroids compared to shCtr.

Given the above results, we investigated whether targeting PTK7 with the mAb cofetuzumab will impair spheroid formation in OC cell lines and PDCs. Cofetuzumab treatment resulted in more disseminated spheroids for JHOS2 and Kuramochi cells (Fig. 5D, Supplementary Fig. 5C) and a similar effect was observed in cofetuzumab-treated FIMM-PDC3 and FIMM-PDC7 cells (Fig. 5E) that could form spheroids more easily compared to other PDCs, probably due to vimentin expression (Supplementary Fig. 5D).

### Targeting PTK7 modulates drug sensitivity in OC cells

Next, we explored how targeting PTK7 in OC cells is reflected in drug response phenotypes by profiling JHOS2, Kuramochi, and Ovsaho and their shPTK7 clones using drug sensitivity and resistance testing (DSRT) screens. In total, we quantified drug sensitivities of 525 approved and investigational oncology compounds over a 10,000-fold concentration range (Supplementary Table 2) and drug responses were quantified as an area under curve and defined as the drug sensitivity scores (DSS), as described before [30]. The cut-off for a significant drug response was defined by a DSS value 8 that represents the 80th percentile of the DSS distribution of all drugs in all samples. (Supplementary Fig. 6A).

**Fig. 6:**
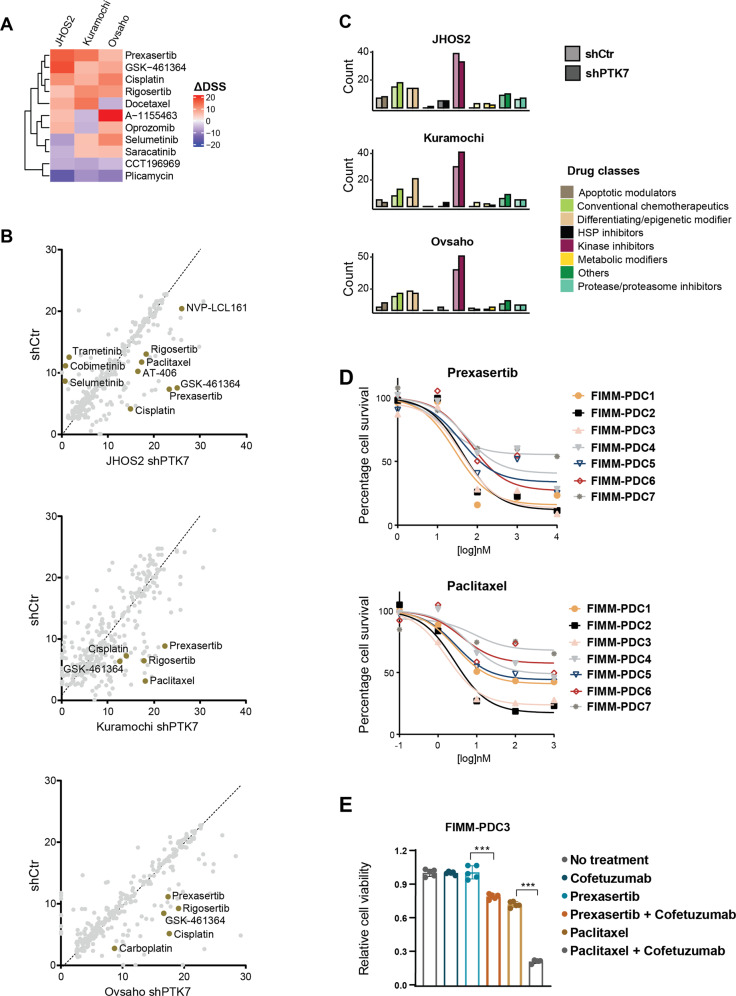
Phenotypic profiling of drug responses following PTK7 receptor targeting. **A** Hierarchically clustered heatmap of common drugs having a ΔDSS ≥ 5 or ≤ −5 in all JHOS2, Kuramochi, and Ovsaho shRNA cell lines. **B** Scatterplot of the DSS values for shCtr (y-axis) and shPTK7 (x-axis) samples in JHOS2, Kuramochi and Ovsaho. The dashed line indicates the path where y = x. Selected drugs are highlighted. **C** Bar chart depicting the count of drugs classified based on their mechanism of action. In (**B**, **C**), only the drugs with DSS ≥ 8 were selected. **D** Cell viability (EC50) curves for prexasertib (CHK1/2 inhibitor) and paclitaxel (taxane agent) in PDCs. **E** Cell viability assays of the FIMM-PDC3 cells treated with 100 nM prexasertib or paclitaxel, and each in combination with 10 µg/ml cofetuzumab. Cells were pre-treated with cofetuzumab (or left untreated) for 24 h before addition of prexasertib or paclitaxel, after which the treatment was continued for 48 h. The values were normalized to the no-treatment control samples and the bar chart shows the mean (*n* = 5) ± SD. The significance between the annotated samples was calculated with two-tailed Student´s *t*-test: ***p ≤ 0.001.

Using the differential drug response values (ΔDSS, cut-off value ≥ 5 and ≤ −5) that defines the gained or lost drug sensitivity after receptor targeting compared to parental DSS for each cell line, we identified increased drug sensitivities for CHK1/2 inhibitor prexasertib, PLK1 inhibitors GSK461364 and rigosertib, and chemotherapeutic agent cisplatin following PTK7 targeting in all cell lines (Fig. 6A). We also noticed loss of sensitivity for kinase inhibitors targeting MEK1/2 (trametinib, cobimetinib, and selumetinib) whereas SMAC mimetics (NVP-LCL161, AT-406, and birinapant) gained sensitivity upon PTK7 targeting in JHOS2 cells (Fig. 6B). In general, we observed that kinase inhibitors gained potency in Kuramochi and Ovsaho cells following PTK7 targeting (Fig. 6C).

We could experimentally validate several of the ΔDSS values identified above. Enhanced sensitivity for SMAC mimetics following PTK7 knockdown was confirmed in a small-scale drug testing in JHOS2 cells (Supplementary Fig. 6B). Previously, we have shown that NF-κB pathway activation could play a role in SMAC mimetics sensitivity in OC cells [17]. However, the activation of NF-κB (as judged by the NF-κB p65 phopsho-Ser536 levels) in our PDCs ex vivo did not always correlate with the high DSSs of the SMAC mimetics in all samples (Supplementary Fig. 6C), suggesting that some other underlying mechanisms define SMAC mimetics sensitivity in clinical samples. We also observed downregulation of pMEK1/2 level in JHOS2 shPTK7 compared to shCtr cells (Supplementary Fig. 6D), which confirmed the loss of MEK1/2 inhibitors sensitivity in these cells.

Looking at drugs with clinical significance for OC treatment, we identified prexasertib and paclitaxel that showed enhanced ΔDSSs following PTK7 targeting (Fig. 6A, B). Prexasertib is a novel CHK1/2 inhibitor under investigation for the treatment of BRCA wild-type HGSC in phase II clinical trial showing good tolerability profile [31], whereas paclitaxel, a taxane agent, is routinely used in OC treatment [32]. Indeed, we observed that prexasertib and paclitaxel showed significant cytotoxicity in OC PDCs, indicative of clinical potential for their synergistic activity with co-targeting PTK7 (Fig. 6D). Treatment of FIMM-PDC3 that expresses high levels of PTK7 with cofetuzumab, either alone or in combination with prexasertib or paclitaxel, resulted in statistically significant enhanced cytotoxicity when both agents were used (Fig. 6E). At the molecular level, immunoblot analysis showed that paclitaxel alone downregulated PTK7 levels in Kuramochi and FIMM-PDC3 (Supplementary Fig. 6E), which is in line with the transcriptomic analysis of DECIDER clinical samples showing downregulated PTK7 levels in post-NACT samples. Interestingly, we observed a stronger downregulation of PTK7 levels following combination of cofetuzumab and paclitaxel than either treatment alone, suggesting that paclitaxel augments the effect of PTK7 mAb. On the other hand, downregulation of β-tubulin, which is the target of paclitaxel, was observed in all paclitaxel-treated samples.

Other drugs with synergistic potential in combinatorial treatments were PLK1 inhibitors GSK-461364 and rigosertib, showing overall good cytotoxicity in some PDCs (Supplementary Fig. 6F). Both drugs have been evaluated in phase I/II clinical trials for solid tumors including some OC patients, supporting further clinical studies in single or combination regimen [33, 34]. Taken together, our DSRT results suggest that combination of other targeted or chemotherapeutic agents to PTK7 targeting may increase the pharmacological efficacy compared to single-agent treatment in OC.

## Discussion

Here, we described transcriptomic, proteomic, phosphoproteomic, and high throughput drug testing datasets that provide a systemic view of the PTK7-regulated signaling network in OC, integrated with ROR1 and ROR2, which share the same Wnt5a ligand. Although this resource enabled the identification of differences between PTK7 and ROR1/2 signaling networks, we observed marked similarity between the phosphoproteomics and phenotypic output of these receptors. Our RNA-seq analysis of HGSC tumor samples identified decreased PTK7 expression in post-NACT samples but the expression was elevated in relapsed samples. Immunoblot analysis of PDCs from type I and type II OC tumors revealed that PTK7 expression was more prevalent compared to ROR1 or ROR2, suggesting that this receptor is more ubiquitously expressed among these non-canonical Wnt receptors.

PTK7 knockdown had a marked impact on JHOS2 cell proliferation in comparison with ROR1 and ROR2 knockdown that did not affect cell survival. Proteomics analyses of JHOS2 shPTK7 samples identified downregulation of PPP1CC/PP1γ, the γ-isoform of PP1 phosphatase involved in the regulation of cell cycle progression by modulating the turnover of key cell-regulatory proteins and responsible for dephosphorylating multiple cellular components [35, 36]. We also noticed that targeting PTK7 with the mAb cofetuzumab appeared to upregulate, albeit at lower levels, ROR1 and pERK/pAKT in some OC cells, indicating that this mechanism could then be linked to PTK7 downregulation. Another downstream PTK7 target was SNCG/synuclein-γ/, a GPCR substrate with functional relevance in OC disease progression [37]. Corroborating these findings, SNCG was highly expressed in OC TCGA data compared to other prevalent cancers in women, and SNCG levels were statistically higher in post-NACT compared to treatment-naïve OC tumors. Functionally relevant, we found that siRNA-mediated downregulation of SNCG or PP1γ resulted in decreased PTK7 levels in OC cell lines, pointing toward a regulatory feedback loop modulating PTK7 and SNCG or PP1γ expression.

Furthermore, we evaluated the functional determinants of these Wnt5a receptors in OC via phosphoproteomics analyses and identified enrichment of several alterations in cancer-associated pathways involved in adhesion, migration, Rho-GTPases, and MAPK pathway following receptors targeting. A dynamic shift in phosphoproteins such as vimentin, δ-catenin, lamin-B1, stathmin-1, and PRPF4B following receptor(s) knockdown points toward their roles as regulators of cytoskeleton plasticity to facilitate cell migration and EMT, as previously suggested [38–42]. In addition, among our top 50 dysregulated phosphoproteins were cortactin and tight-junction protein-family protein (TJP2, or ZO-2) as well as AHNAK1, known to be involved in tumor metastasis through EMT [43, 44]. Our HCiA clearly showed that targeting PTK7 inhibits spheroid formation, and a similar outcome was observed in cofetuzumab-treated OC cells. Although our findings are consistent with other reports showing that both ROR1 and ROR2 are involved in cell motility and Rho-GTPases activation in cancer cells [17, 45, 46], we provide evidence that PTK7 also participates in similar processes in OC cells to sustain disease progression via cell state plasticity and adhesion dynamics. EMT is regarded as a critical step for OC progression being significantly associated with peritoneal metastasis and patient survival [47, 48]. Previous studies have also demonstrated that metastatic OC tumors generally exhibit mesenchymal phenotype [49], and chemoresistant OC cells frequently display mesenchymal traits [50]. Our data offers clear evidence that targeting PTK7 expression impairs migration, EMT, and spheroid formation in OC that will likely interfere with disease progression.

The complex heterogeneity of OC represents a substantial challenge for therapeutic progress, and our analysis of DSRT data revealed insight into PTK7 pseudokinase mechanisms-of-action that could be clinically valuable. Currently, PTK7 mAb-ADC cofetuzumab-pelidotin has entered clinical trials for solid tumors, including OC (NCT02222922) [11] indicating that this receptor offers excellent opportunities as clinical target. Our study suggests that targeting PTK7 could augment drug responses for both targeted drugs (prexasertib, rigosertib, GSK461364, SMAC mimetics, among others) and chemotherapeutic agents such as paclitaxel, and that these responses are dependent on the expression levels of PTK7 as well as the underlying pharmacogenomic profile linked to OC subtypes. In line with this, PTK7 was more commonly expressed than ROR1/2 in our PDCs, and HGSC subtype showed higher PTK7 expression than LGSC. We also show that in FIMM-PDC3 OC primary sample that has high levels of PTK7 there is a synergistic mechanism between cofetuzumab and prexasertib or paclitaxel treatments, which supports the in vitro data from shRNA screens. This synergistic effect of targeting a RTKs family member (with an antibody-based molecule) in combination with paclitaxel (Taxol) has shown better efficacy in many preclinical and clinical settings [51, 52]. The rationale would be to target two complementary pathways, the RTKs signaling and the microtubule/cytoskeleton assembly. The downregulation of PTK7 by paclitaxel is clinically relevant, as it would explain the decreased PTK7 levels in post-NACT samples compared to treatment-naïve samples. Consequently, the efficacy of PTK7 mAb in post-NACT samples could be compromised due to lower PTK7 levels, which prompts for a careful selection of patients for targeted PTK7-based clinical treatments.

Taken together, our present data brings novel mechanistic insight into understanding the role of non-canonical Wnt signaling via PTK7 in promoting OC disease progression. Moreover, the study provides new conceptual information for therapeutic development of treatment strategies via (co-)targeting this pseudokinase.

## Supplementary information

Supplementary Materials and Methods

Supplementary Figure 1

Supplementary Figure 2

Supplementary Figure 3

Supplementary Figure 4

Supplementary Figure 5

Supplementary Figure 6

Supplementary Figure 7, Uncropped western blots

Supplementary Table 1

Supplementary Table 2

Checklist

## Data Availability

All data relevant to the study are included in the article or uploaded as supplementary information and are available on reasonable request from the corresponding author.

## Code availability

All data relevant to the study are included in the article or uploaded as supplementary information and are available on reasonable request from the corresponding author.
