# Supplementary material for: Multiomics characterization implicates PTK7 in ovarian cancer EMT and cell plasticity and offers strategies for therapeutic intervention
Source: Cell Death Dis. 2022 Aug 17;13(8):714. doi: 10.1038/s41419-022-05161-5 (PMC9386025; doi:10.1038/s41419-022-05161-5)
Supplement: Supplementary file 1 — Supplementary Materials and Methods [file 41419_2022_5161_MOESM1_ESM.docx]

**Supplementary Materials and Methods**

## **Cell culture**

Ovarian cancer cell lines Kuramochi and Ovsaho were obtained from JCBR cell bank and grown in RPMI 1640 media (Lonza, Basel, Switzerland) supplemented with 10 % fetal bovine serum, 2mM L-glutamine, and Primocin™ (InvivoGen, San Diego, CA, USA). JHOS2 cells were obtained from Riken BRC and grown in DMEM/F-12 media (Gibco™, Thermo Fisher Scientific, Waltham, MA, USA) supplemented with 10 % fetal bovine serum, 1xMEM non-essential amino acid solution (Thermo Fisher Scientific, Waltham, MA, USA) and Primocin™. Cell counting was done with Countess II automated cell counter (Invitrogen™). Stable shRNA cell lines expressing control-, ROR1-, ROR2-, and PTK7- shRNA were generated as described previously [1,2] by using an inducible pLKO-Tet-On lentiviral expression system. The expression of shRNA was induced by 100 ng/ml doxycycline (DOX) treatment for at least three days and ROR1/ROR2/PTK7 silencing was verified by immunoblot analysis. Two shRNAs were used for each target and the isogenic clones with the most efficient knockdown were used for further functional analysis. To target PTK7 expression with monoclonal antibody, the cells were treated with cofetuzumab (AH784, Geneva Antibody Center, Switzerland) for various assays as indicated.

## **Cell viability assay**

CellTiter-Glo (CTG) 2.0 Assay (Promega, Madison, WI, USA) was used to measure cell viability accordingly to manufacturer’s instruction. Luminescence signal was detected using Envision plate reader (PerkinElmer, Waltham, MA, USA) or PHERAstar FS (BMG Labtech, Ortenberg, Germany).

Cell viability of the spheroids was detected according to the manufacturer’s protocol using CellTiter-Glo 3D Reagent (Promega) and the luminescence was measured with PHERAstar FS (BMG).

## **Immunoblotting**

Cells were lysed with Triton-X lysis buffer (50 mM Tris-HCl pH 7.5, 10 % glycerol, 150 mM NaCl, 1 mM EDTA, 1 % Triton-x-100, 50 mM NaF) supplemented with protease and phosphatase inhibitor cocktails (Pierce Protease and Phosphatase Inhibitor Mini Tablets, Thermo Fisher Scientific). Lysates were mixed with Laemmli sample buffer (Bio-Rad Laboratories, California, USA), separated in SDS-PAGE, and transferred to nitrocellulose membranes, followed by incubation with the following primary antibodies (all used 1:1 000 dilution, if not stated otherwise): β-tubulin (sc-166729, Santa Cruz, Dallas, TX, USA), PP1CC (sc-515943, Santa Cruz; 1:200 dilution) β-actin (#3700, Cell Signaling Technology (CST), Danvers, MA, USA), AKT (#2920, CST), AKT-pSer473 (#4060, CST), E-cadherin (#14472S, CST), E-cadherin (#3195, CST), ERK1/2 (#4696, CST), ERK1/2-pThr202/Tyr204 (#9101, CST), MEK (#4694, CST), MEK1/2-pSer217/221 (#9121, CST), NF-κB p65 (#6956, CST), NF-κB p65-pSer536 (#3033, CST), PAX8 (#10336-1-A, Proteintech, Manchester, UK), PTK7 (#25618, CST), ROR1 (#16540, CST), ROR2 (#88639, CST), SNCG (ab52633, Abcam, used 1:500 dilution), Vimentin (#5741, CST), Wnt5a (#2530, CST). Secondary antibodies were IRDye® 800CW Donkey anti-Mouse IgG or IRDye® 680RD Donkey anti-Rabbit IgG (LI-COR, Lincoln, NE, USA) and blots were scanned with Odyssey® Fc Imaging system (LI-COR). Image analysis was done using the Image Studio Lite software (LI-COR). Whole blots of the figures are in Supplementary Fig. 7.

## **Spheroid formation assay**

Cell lines were cultured 4-6 days in MBEM media (Misvik Biology, Turku, Finland) supplemented with 2% FBS, 2 mM L-glutamine, and Primocin™ on ultra-low attachment plates (Corning incorporated, NY, USA). PDCs were cultured in their specific media. Images were taken with Invitrogen™ EVOS™ FL imaging system (Thermo Fisher Scientific) or Leica DMI LED microscope (Leica microsystems, Wetzlar, Germany) with Nikon DS-fi1 camera (Nikon, Tokyo, Japan).  The shRNA JHSO2 cells were induced with 100 ng/ml DOX for three days prior to setting up the matrigel-grown spheroids on a 96-well plate in 100% Cultrex^TM^ (R&D Systems, Inc., Minneapolis, MN, USA) and immersed with MBEM media (Misvik Biology) supplemented with 2% FBS, 2 mM L-glutamine, and Primocin^TM^ and DOX (150 ng/ml). Media was changed and DOX refreshed every four days.

## **Immunofluorescence**

Spheroids were stained live by addition of 25 nM Hoechst (Thermo Fisher Scientific) and 50 nM TMRM (Abcam, Cambridge, UK) into the cells for 24-48 h and imaged with EVOS™ FL imaging system (Thermo Fisher Scientific). JHOS2 and Kuramochi shRNA spheroids were induced with 100 ng/ml DOX for 7 days (DOX was refreshed after 3 days), and at day four 75 nM of TMRM (Tetramethylrhodamine, Methyl Ester, Perchlorate, Thermo Fisher Scientific) was added together with NucBlue (Thermo Fisher Scientific) (0.4 X from the stock concentration) and Dioc6(3) (Thermo Fisher Scientific) (final concentration 0.05 μM). Immunofluorescence signal was read after three days using Opera Phenix® Plus High Content Screening System 20X objective. Raw image correction, object identification and morphological quantification were conducted in Harmony (Perkin Elmer).

**siRNA transfection**

The ON-TARGET*plus* siRNA constructs (Dharmacon, CO, USA) against PP1γ, SNCG and Ctr (scrambled) were transfected to JHOS2 and Kuramochi cells seeded into 6-well plates, as per the manufacturer’s protocol, using DharmaFECT™ transfection reagent 1. The transfection was allowed to continue for 72 h before lysing the cells and performing immunoblot analysis to detect the protein levels.

**Wound-healing assay**

JHOS2 and Kuramochi shCtr and shPTK7 cells were induced with 100 ng/ml DOX for 6 days (refreshed every 3th day) before adding 50 nM TMRM. After 24 h a wound was created, and the healing was observed every 24 h with Invitrogen™ EVOS™ FL imaging system (Thermo Fisher Scientific). The width of the wound (in pixels) was analyzed with ImageJ [3] wound-healing size tool, and the significance was calculated from 3 or 4 separate images taken from biological replicates.

**Drug sensitivity and resistance testing (DSRT)**

DSRT (drug sensitivity and resistance testing) was done as described previously [4,5]. Briefly, 1 000 – 1 500 cells were added to wells of 384-well plates with drugs pre-plated over a 10,000-fold concentration range (in five concentrations). After a three-day incubation at 37 °C and 5.0 % CO_2_, cell viability was measured using the Cell Titer-Glo reagent (Promega) and luminescence was read with PHERAstar FS (BMG Labtech). For each drug, dose response curves were generated and DSS were calculated as previously described [4,5]. DSRT profiles of parental JHOS2, Kuramochi and Ovsaho cell lines were identical with their respective shCtr. In the case of the hierarchically clustered heatmap (Euclidean distance, complete linkage) for ΔDSS, an additional cut-off ≥ 5 and ≤ -5 was considered for each cell line individually.

**Proteomics analyses**

### Protein sample preparation

Protein concentrations were measured from thawed cell lysates using a BCA protein assay kit (Thermo Scientific). 300 µg of protein was then taken from each sample, and sample volumes were adjusted to 300µl 8M urea on ice. Cysteine bonds were reduced with 5 mM Tris(2-carboxyethyl) phosphine (TCEP) for 30 minutes at 37 °C, followed by alkylation with 10 mM iodoacetamide for 20 minutes in the dark. 1 ml of 50 mM AMBIC was then added to neutralize the urea buffer. Proteins were digested to peptides with sequencing grade modified trypsin (Promega V5113), at 1:100 enzyme:protein ratio at 37 °C overnight. The following day, pH of the samples was adjusted to < 3 with TFA, and ACN was added to a final concentration of 1 % (v/v). Samples were desalted with BioPureSPN Macro Desalting columns (The Nest Group, Inc, Ipswich, MA, USA). The columns were conditioned with 200 µl of ACN and centrifuged for 1 min at 55 g, followed by flushing with 200 µl of MQ water and 1 min 55 g centrifugation. Column was then equilibrated with 200 µl of buffer A (0.1 % TFA, 1 % ACN), after which the samples were processed 200 µl at a time, followed by two washes with buffer A. Finally, samples were released with 200 µl of elution buffer (80 % ACN, 0.1 % TFA). The elution step was repeated three times, after which the samples were split to total proteome (50 µg) and phosphoenrichment (250 µg) samples and dried in a vacuum centrifuge.

### Phosphopeptide enrichment

Phosphopeptide enrichment was done using immobilized metal ion affinity chromatography with Ti4+-IMAC microspheres. The IMAC material was prepared by following the protocol published previously [6], and for the enrichment of phosphopeptides, the IMAC beads were loaded onto StageTips (Thermo Fisher Scientific), and the material was conditioned as previously described previously [7].

### LC-MS/MS Analysis

The LC-MS/MS analysis was performed, and the spectra acquired as described in [7]. For the total proteome samples, three biological replicates were used, and for each run, 2 µl of sample was injected.

### Database searches

For the phosphopeptide samples, LC-MS/MS analysis was performed as before [7], except the injection volume was set to 8 µl. The raw data files were processed with MaxQuant (version 1.6.4.3 [8]), and the MS spectra were searched, modified and filtered as described in [7]. The produced phosphosite data was analyzed manually and the identified phosphosites were filtered based on localization probability with a cut-off at 0.75. GO enrichment analysis of the phosphopeptides was performed with DAVID [9,10] using the full list of phosphoproteins in each sample. Dysregulated phosphoproteins were identified by calculating the difference in phosphocounts between each shRNA cell line and the respective control for JHOS2, Kuramochi, and Ovsaho separately. Pathway-oriented over-representation analysis was performed with ConsensusPathDB [11] for the top 50 dysregulated phosphoproteins with the highest abundance of phospho-serine and phospho-threonine counts, together with all the hits for phospho-tyrosine counts. Out of 110 proteins, 100 were mapped correctly in the database; 82 were present in at least one pathway, and 84 enriched pathway-based sets were identified (*p* < 0.05*,* false discovery rate (FDR)-adjusted). The results were further validated through over-representation analysis with GeneTrail [12] and STRING [13] for ROR1, ROR2, and PTK7 taken individually or in combination against the following databases: WikiPathway, KEGG and Reactome (*p* < 0.05, FDR-adjusted).

## **Clinical samples**

The sample collection and analysis are part of the DECIDER cohort (https://www.deciderproject.eu/). The sample cohort consists of 82 patients that were treated for high grade serous ovarian cancer (HGSC) at Turku University Hospital between November 2011 and August 2021 (diagnose time: November 2011 to October 2019). All patients participating in the study provided written informed consent. The study and the use of all clinical material have been approved by The Ethics Committee of the Hospital District of Southwest Finland (ETMK) under decision number EMTK: 145/1801/2015. From the cohort, 125 intra-abdominal peritoneal or omental metastatic samples were collected. Of these, 75 were treatment-naïve, 46 were post-NACT, and 4 were relapsed tumor samples.

**Fig. 1A, Fig. 3A, E** The TCGA RNA-seq data for ovarian cancer (OC), breast invasive carcinoma (BRCA), colorectal adenocarcinoma (COAD), uterine corpus endometrial carcinoma (UCEC), lung adenocarcinoma (LUAD), cervical and endocervical cancers (CESC) and skin cutaneous melanoma (SKCM) were downloaded from TCGA using TCGABiolinks (version 2.22.2 [14]) as HTseq gene level effective counts. Counts were converted to transcripts per million (TPM) with GeoTcgaData (version 1.0.2 [15]) and transformed to log2(TPM + 1). Gene symbols were retrieved from the correspondent Ensembl gene IDs using EnsDb.Hsapiens.v79 (version 2.99.0 [16]). All analyses were performed using the R software (version 4.1.1 [17]). **Fig. 2** For the differential protein expression analyses of total proteome samples, the DEP R package (version 1.16.0 [18]) was adopted. The fold-change cut off for differentially expressed proteins (DEPs) was set to 1, and the *p*-value (*p*) corresponds to FDR-adjusted *p*-value for multiple testing. **Fig. 5C** The Uniform Manifold Approximation and Projection (UMAP) for JHOS2 and Kuramochi spheroid imaging data for Fig. were obtained using the R package umap (version 0.2.70 [19]).

**Fig. 1C, Fig. 3B, F** Bulk RNA sequencing reads from DECIDER were preprocessed using the SePIA pipeline [20] within the Anduril framework [21]. Read pairs were trimmed using Trimmomatic (version 0.33 [22]) and aligned to the GRCh38.d1.vd1 reference genome with GENCODE v25 annotation using STAR (version 2.5.2b [23]). Gene level effective counts (found to be more accurate than the raw read counts) were quantified using eXpress (version 1.5.1-linux_x86_64 [24]). The batch effects between different library preparation protocols were removed using POIBM (<https://bitbucket.org/anthakki/poibm/>). Cell type specific expression profiles for cancer (epithelial) cells from the bulk RNA-seq data were deconvoluted with PRISM [25].

**Supplementary Fig. 1A, Supplementary Fig. 3A** Data for tumoral and normal tissue samples were retrieved from TCGA and the Genotype-Tissue Expression (GTEx) via the Xena platform available at UCSC (http://xena.ucsc.edu/) for the following primary tumor sites [26]: ovary (TCGA *n* = 418, GTEx normal fallopian tube tissue *n* = 5), breast (TCGA *n* = 1205, GTEx *n* = 179), lung (TCGA *n* = 1 120, GTEx *n* = 287), colon (TCGA *n* = 329, GTEx *n* = 304), cervix (TCGA *n* = 307*,* GTEx *n* = 10), uterus (TCGA *n* = 57, GTEx *n* = 78), and skin (TCGA *n* = 103, GTEx *n* = 556). **Supplementary Fig. 2** ROR1 and PTK7 staining for flow cytometry was done with anti-ROR1-PE 2A2 antibody and PTK7-APC antibody (Miltenyi Biotec, Bergisch Gladbach, Germany), respectively, according to manufacturer’s instructions. Cell detaching was done with Accutase® cell detachment solution (Innovative Cell Technologies, San Diego, CA, USA). Flow cytometry samples were run with BD Accuri C6 (BD Biosciences, San Jose, CA, USA) and analyzed with BD Accuri C6 software.

**References:**

1. Wiederschain D, Wee S, Chen L, Loo A, Yang G, Huang A, et al. Single-vector inducible lentiviral RNAi system for oncology target validation. Cell cycle (Georgetown, Tex). 2009 Feb;8(3):498–504.

2. Karvonen H, Niininen W, Murumägi A, Ungureanu D. Targeting ROR1 identifies new treatment strategies in hematological cancers. Biochemical Society Transactions. 2017 Apr 15;45:457–64.

3. Schneider CA, Rasband WS, Eliceiri KW. NIH Image to ImageJ: 25 years of image analysis. Nature Methods. 2012;9(7):671–5.

4. Pemovska T, Kontro M, Yadav B, Edgren H, Eldfors S, Szwajda A, et al. Individualized systems medicine strategy to tailor treatments for patients with chemorefractory acute myeloid leukemia. Cancer discovery. 2013 Dec;3(12):1416–29.

5. Yadav B, Pemovska T, Szwajda A, Kulesskiy E, Kontro M, Karjalainen R, et al. Quantitative scoring of differential drug sensitivity for individually optimized anticancer therapies. Scientific Reports. 2014;4(1):5193.

6. Zhou H, Ye M, Dong J, Corradini E, Cristobal A, Heck AJR, et al. Robust phosphoproteome enrichment using monodisperse microsphere-based immobilized titanium (IV) ion affinity chromatography. Nature protocols. 2013 Mar;8(3):461–80.

7. Salokas K, Öhman T, Liu X, Chowdhury I, Gawriyski L, Keskitalo S, et al. Physical and functional interactome atlas of human receptor tyrosine kinases. bioRxiv. 2021;

8. Cox J, Mann M. MaxQuant enables high peptide identification rates, individualized p.p.b.-range mass accuracies and proteome-wide protein quantification. Nature Biotechnology. 2008;26(12):1367–72.

9. Sherman BT, Hao M, Qiu J, Jiao X, Baseler MW, Lane HC, et al. DAVID: a web server for functional enrichment analysis and functional annotation of gene lists (2021 update). Nucleic Acids Research. 2022 Mar 23;gkac194.

10. Huang DW, Sherman BT, Lempicki RA. Systematic and integrative analysis of large gene lists using DAVID bioinformatics resources. Nature Protocols. 2009;4(1):44–57.

11. Kamburov A, Stelzl U, Lehrach H, Herwig R. The ConsensusPathDB interaction database: 2013 update. Nucleic Acids Research. 2013 Jan 1;41(D1):D793–800.

12. Stöckel D, Kehl T, Trampert P, Schneider L, Backes C, Ludwig N, et al. Multi-omics enrichment analysis using the GeneTrail2 web service. Bioinformatics. 2016 May 15;32(10):1502–8.

13. Jensen LJ, Kuhn M, Stark M, Chaffron S, Creevey C, Muller J, et al. STRING 8--a global view on proteins and their functional interactions in 630 organisms. Nucleic acids research. 2009 Jan;37(Database issue):D412-6.

14. Colaprico A, Silva TC, Olsen C, Garofano L, Cava C, Garolini D, et al. TCGAbiolinks: an R/Bioconductor package for integrative analysis of TCGA data. Nucleic acids research. 2016 May;44(8):e71.

15. Hu E. GeoTcgaData: Processing various types of data on GEO and TCGA. R package version 1.0.2. 2021.

16. Rainer J. EnsDb.Hsapiens.v79: Ensembl based annotation package. R package version 2.99.0. 2017.

17. Team RC. R: A language and environment for statistical computing. R Foundation for Statistical Computing. Vienna, Austria. 2021;

18. Zhang X, Smits AH, van Tilburg GB, Ovaa H, Huber W, Vermeulen M. Proteome-wide identification of ubiquitin interactions using UbIA-MS. Nature protocols. 2018 Mar;13(3):530–50.

19. Konopka T. umap: Uniform Manifold Approximation and Projection. R package version 0.2.7.0. 2020;

20. Icay K, Chen P, Cervera A, Rantanen V, Lehtonen R, Hautaniemi S. SePIA: RNA and small RNA sequence processing, integration, and analysis. BioData Mining. 2016;9(1):20.

21. Cervera A, Rantanen V, Ovaska K, Laakso M, Nuñez-Fontarnau J, Alkodsi A, et al. Anduril 2: upgraded large-scale data integration framework. Bioinformatics. 2019 Oct 1;35(19):3815–7.

22. Bolger AM, Lohse M, Usadel B. Trimmomatic: a flexible trimmer for Illumina sequence data. Bioinformatics. 2014;30(15):2114–20.

23. Dobin A, Davis CA, Schlesinger F, Drenkow J, Zaleski C, Jha S, et al. STAR: ultrafast universal RNA-seq aligner. Bioinformatics (Oxford, England). 2013 Jan;29(1):15–21.

24. Roberts A, Pachter L. Streaming fragment assignment for real-time analysis of sequencing experiments. Nature methods. 2013 Jan;10(1):71–3.

25. Häkkinen A, Zhang K, Alkodsi A, Andersson N, Erkan EP, Dai J, et al. PRISM: recovering cell-type-specific expression profiles from individual composite RNA-seq samples. Bioinformatics. 2021 Sep 15;37(18):2882–8.

26. Goldman MJ, Craft B, Hastie M, Repečka K, McDade F, Kamath A, et al. Visualizing and interpreting cancer genomics data via the Xena platform. Nature Biotechnology. 2020;38(6):675–8.
