# Supplementary figures and images for: Multiomics characterization implicates PTK7 in ovarian cancer EMT and cell plasticity and offers strategies for therapeutic intervention
Source: Cell Death Dis. 2022 Aug 17;13(8):714. doi: 10.1038/s41419-022-05161-5 (PMC9386025; doi:10.1038/s41419-022-05161-5)

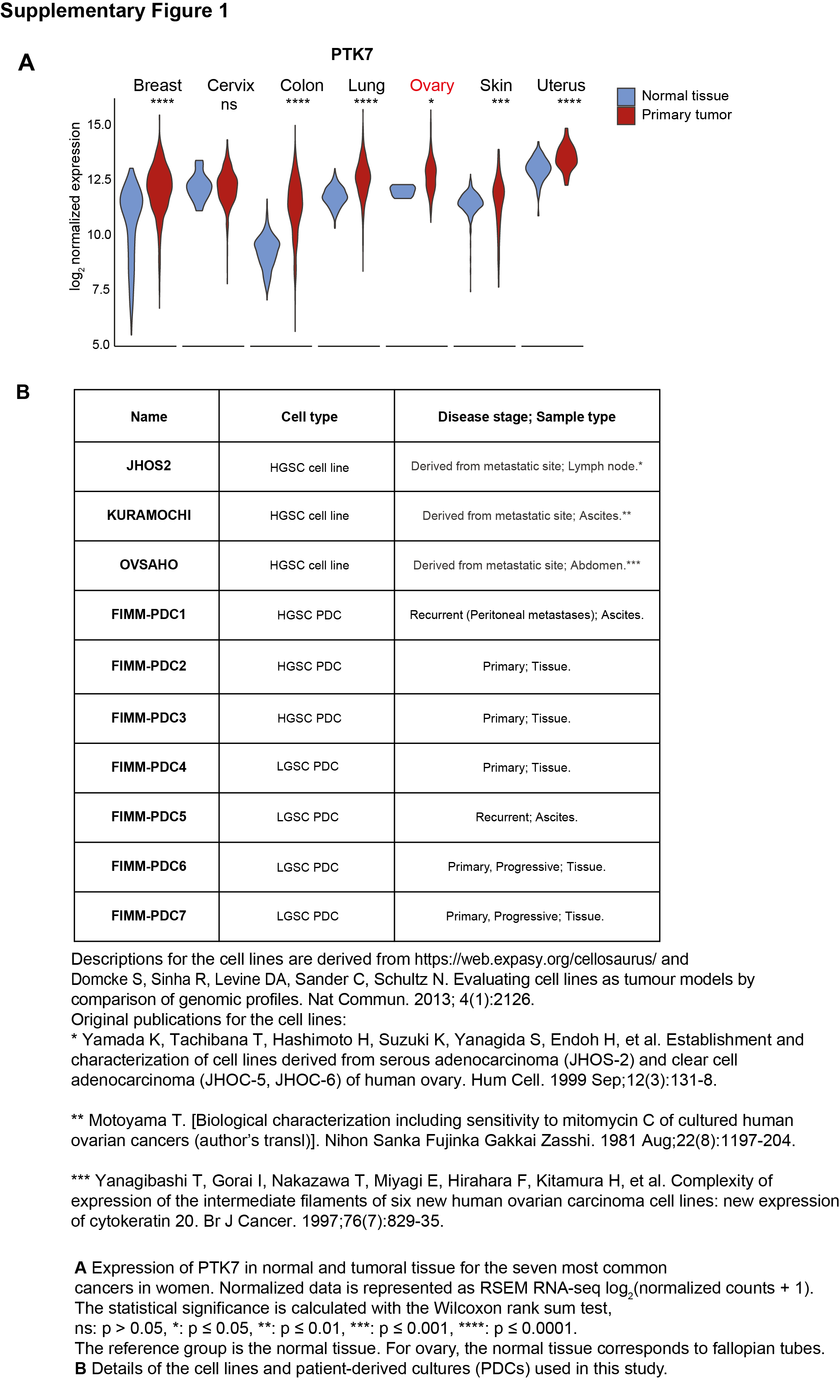

Supplement: Supplementary file 2 — Supplementary Figure 1 [file 41419_2022_5161_MOESM2_ESM.png]

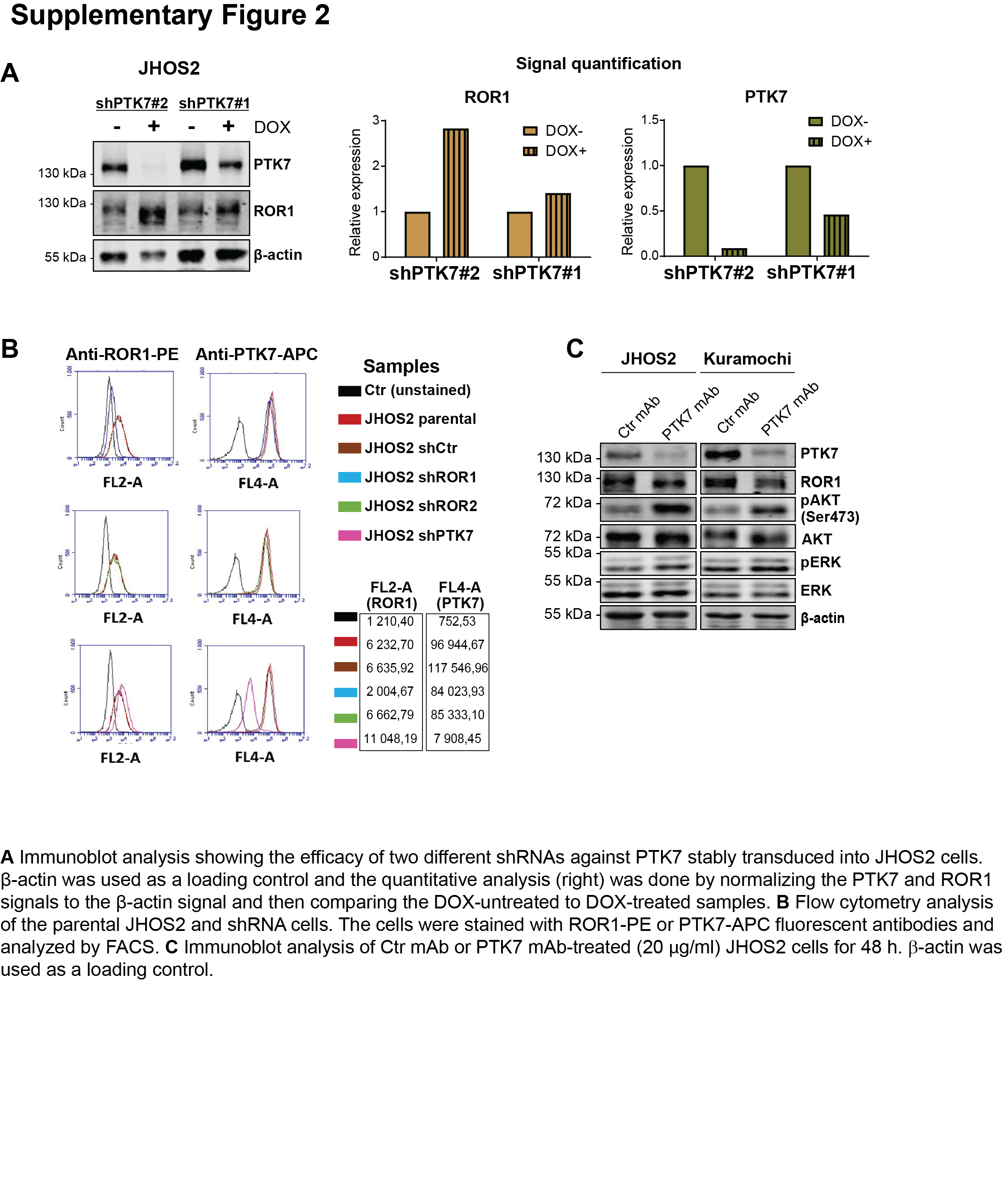

Supplement: Supplementary file 3 — Supplementary Figure 2 [file 41419_2022_5161_MOESM3_ESM.png]

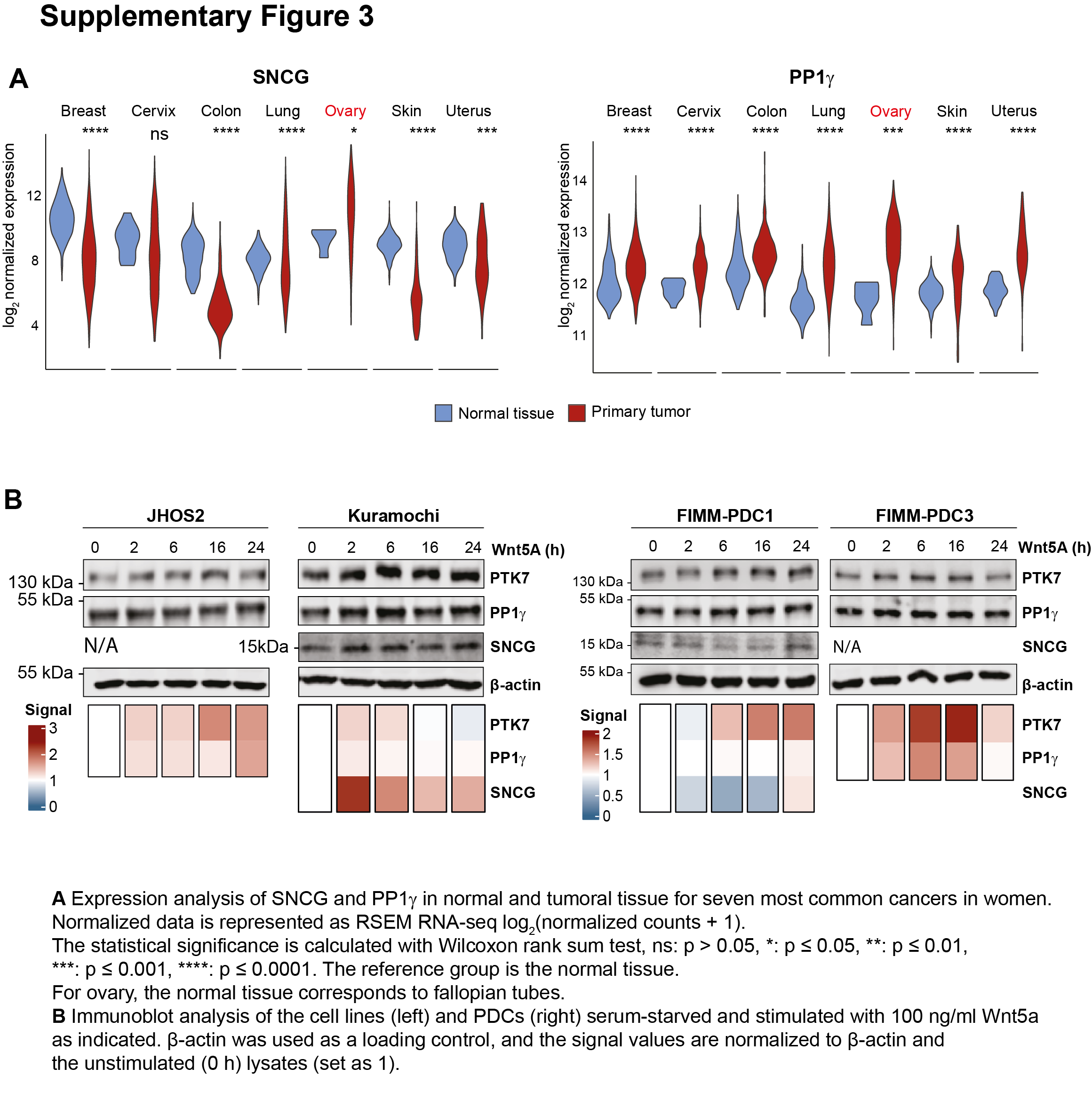

Supplement: Supplementary file 4 — Supplementary Figure 3 [file 41419_2022_5161_MOESM4_ESM.png]

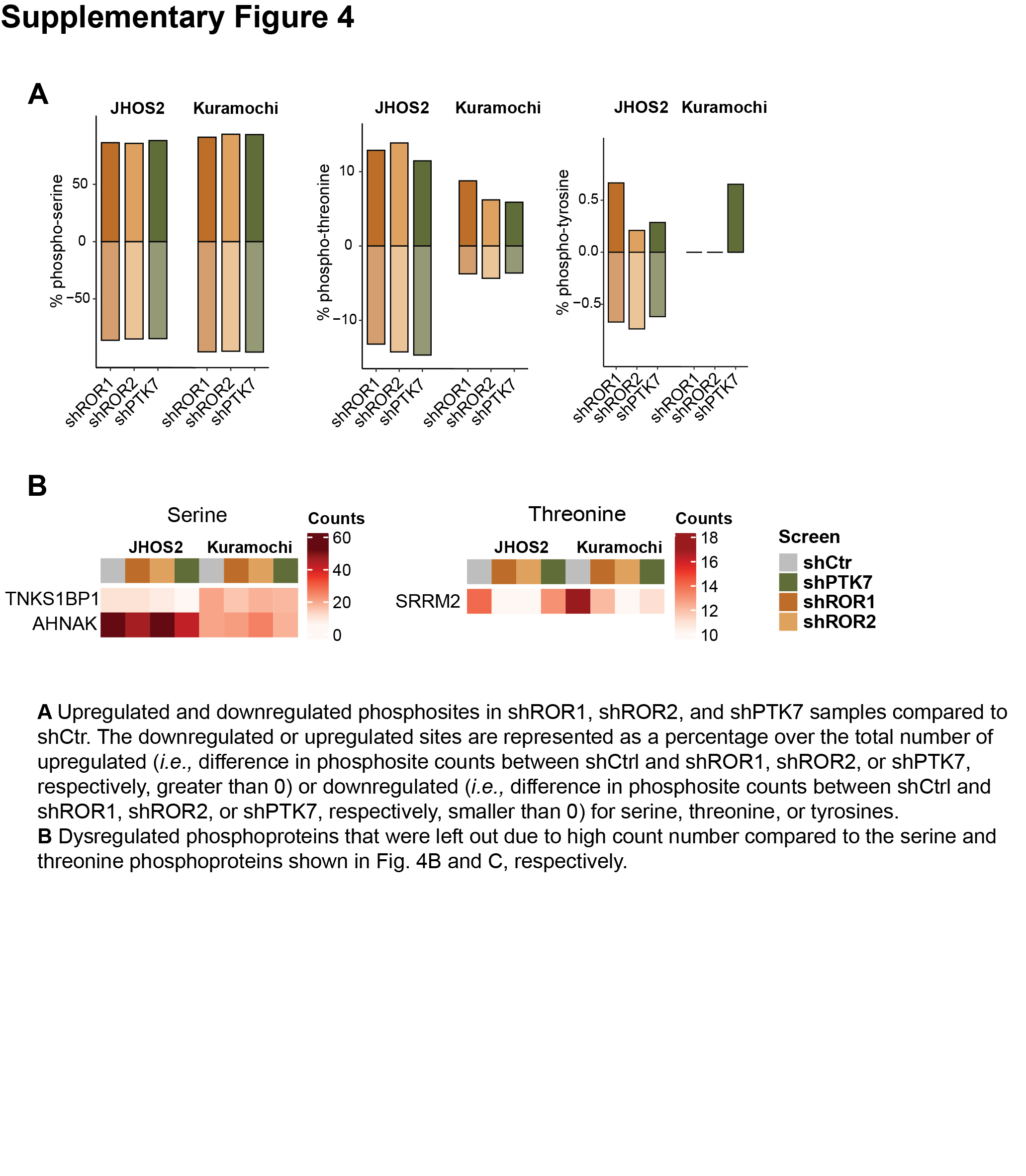

Supplement: Supplementary file 5 — Supplementary Figure 4 [file 41419_2022_5161_MOESM5_ESM.png]

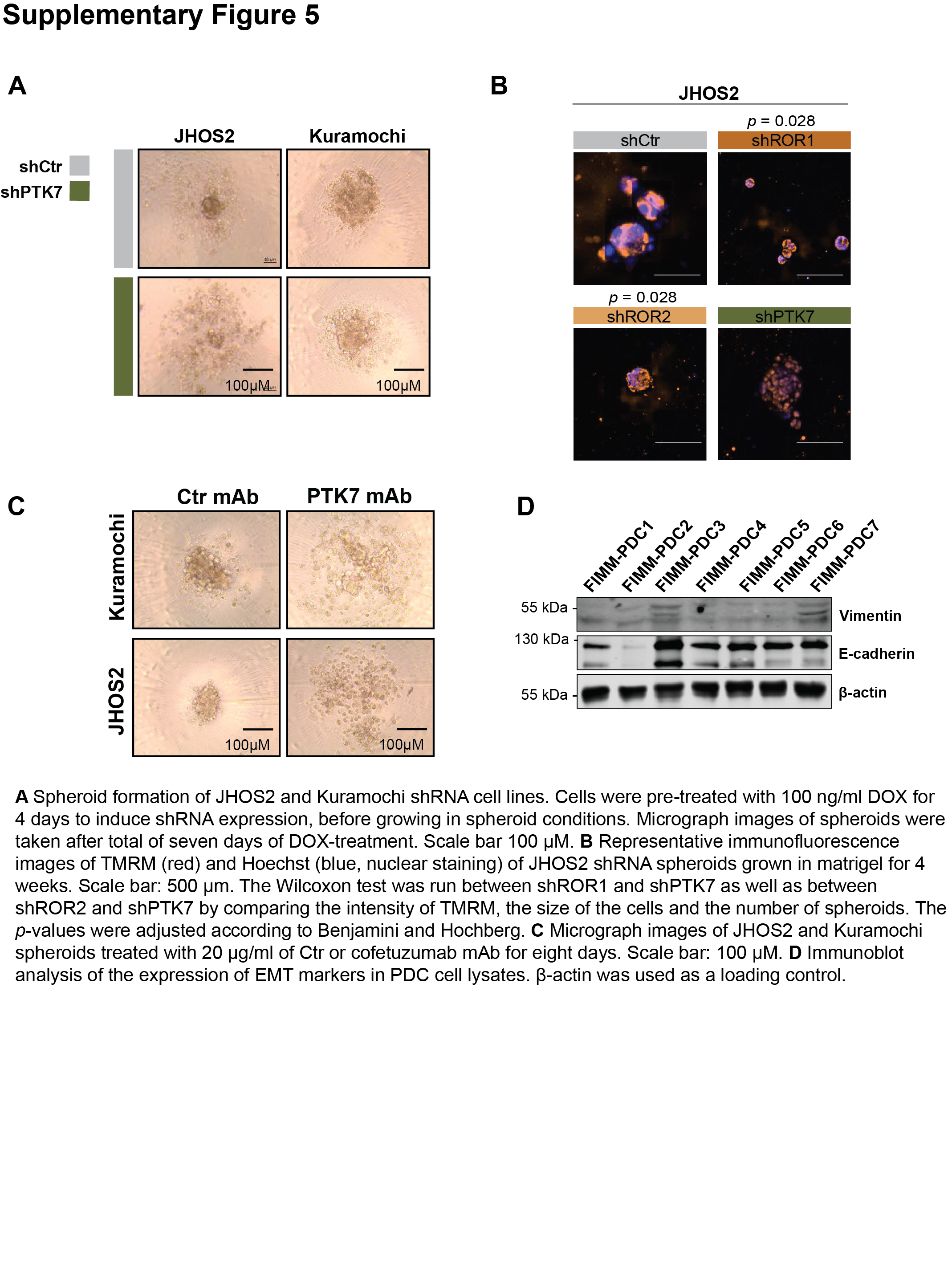

Supplement: Supplementary file 6 — Supplementary Figure 5 [file 41419_2022_5161_MOESM6_ESM.png]

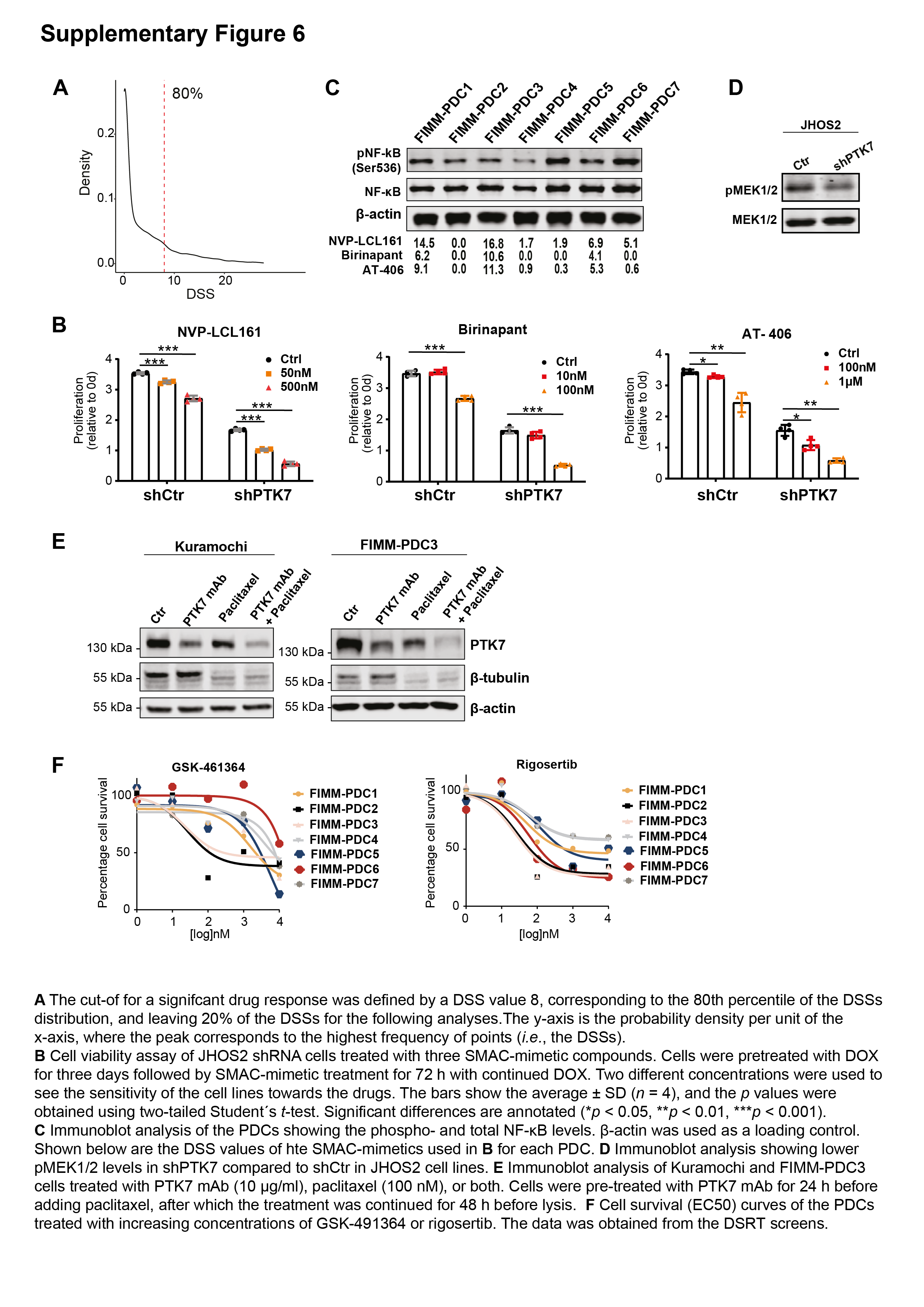

Supplement: Supplementary file 7 — Supplementary Figure 6 [file 41419_2022_5161_MOESM7_ESM.png]
