# Supplementary material for: Multiomics characterization implicates PTK7 in ovarian cancer EMT and cell plasticity and offers strategies for therapeutic intervention
Source: Cell Death Dis. 2022 Aug 17;13(8):714. doi: 10.1038/s41419-022-05161-5 (PMC9386025; doi:10.1038/s41419-022-05161-5)
Supplement: Supplementary file 8 — Supplementary Figure 7, Uncropped western blots [file 41419_2022_5161_MOESM8_ESM.pdf]

Figure 1

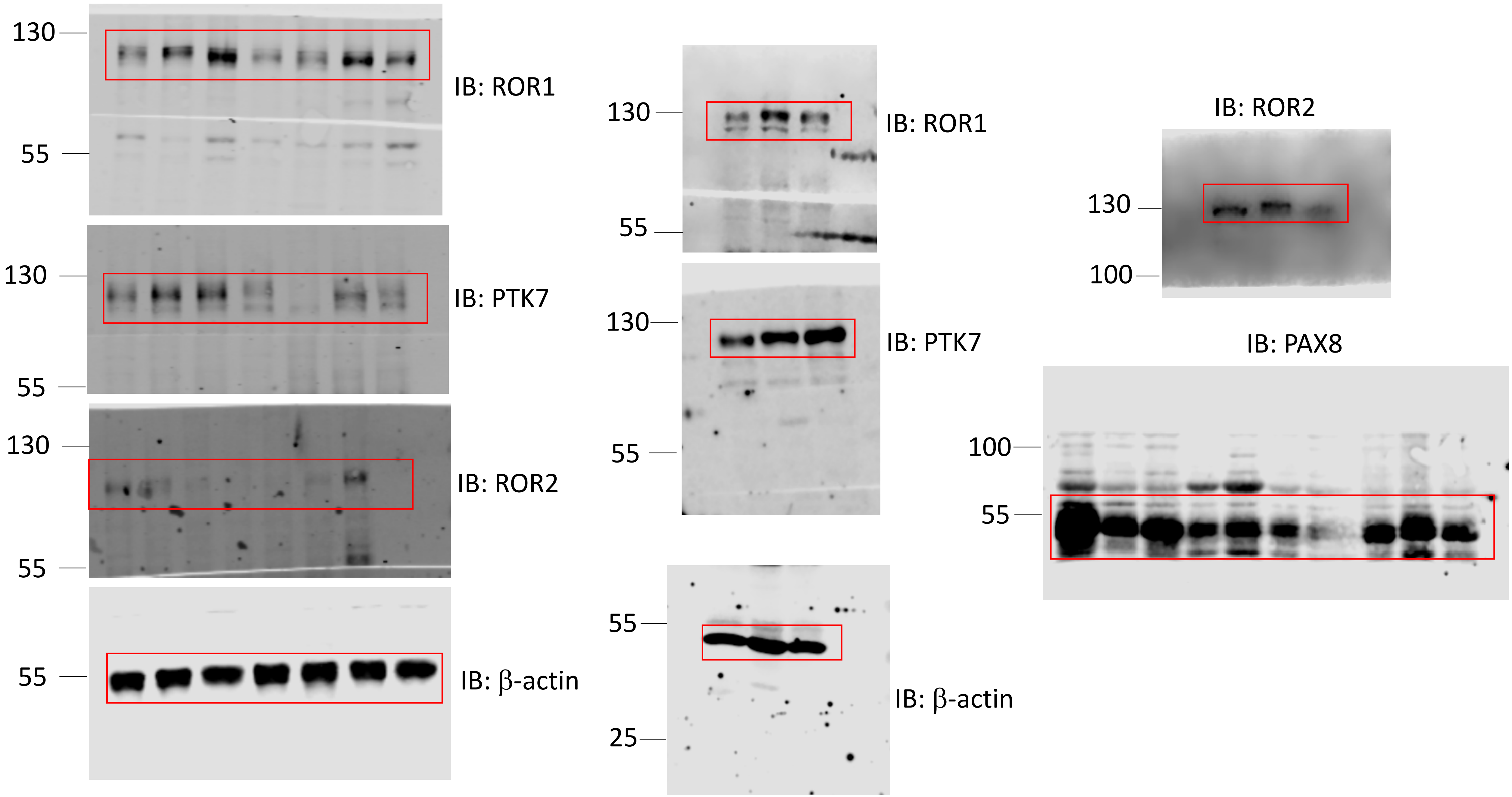

130
55
IB: ROR1
130
55
IB: PTK7
130
55
IB: ROR2
55
IB: β-actin
130
55
IB: ROR1
130
55
IB: PTK7
55
25
IB: β-actin
IB: ROR2
130
100
IB: PAX8
100
55

Figure 2

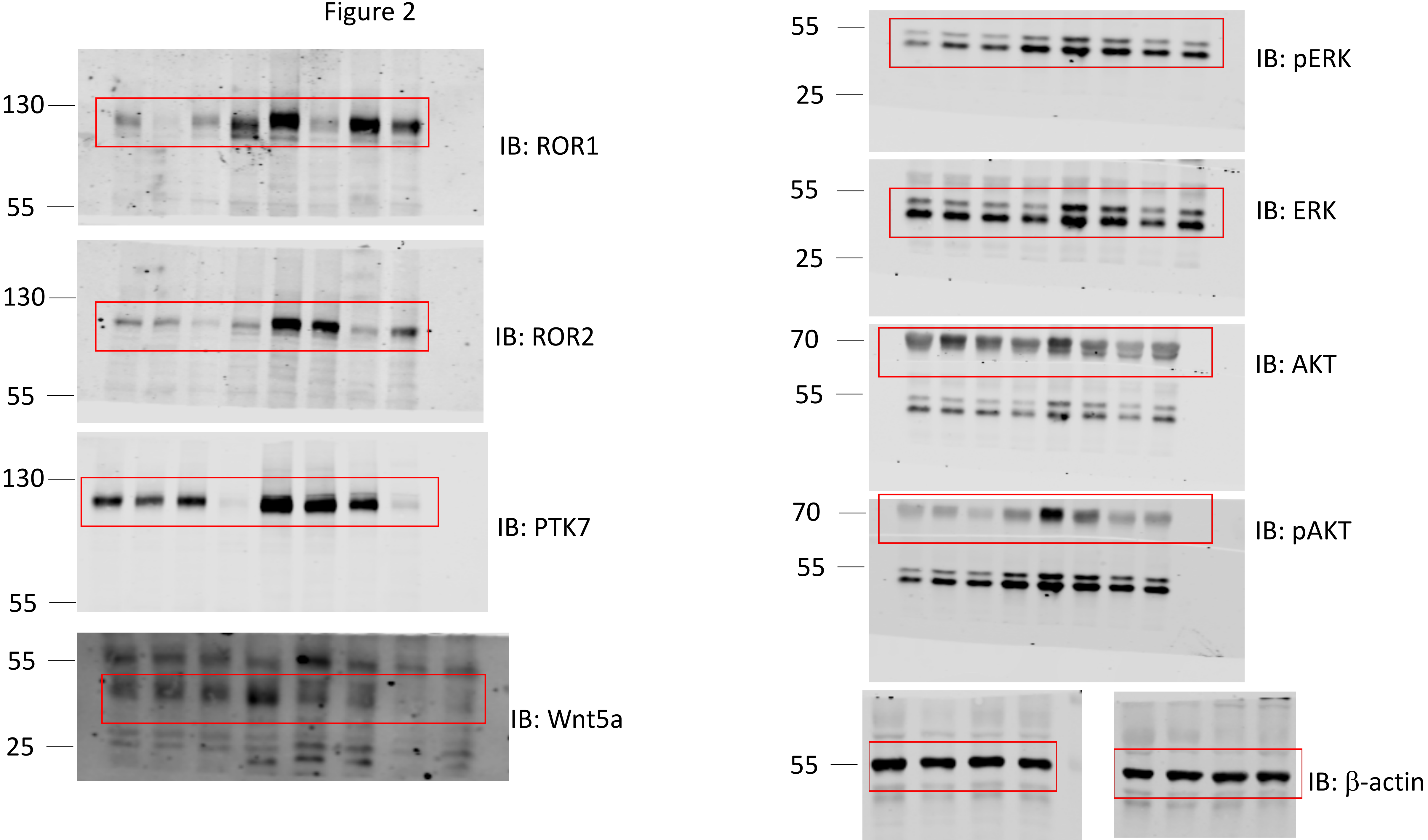
130
55
IB: ROR1
130
55
IB: ROR2
130
55
IB: PTK7
55
25
IB: Wnt5a
55
25
IB: pERK
55
25
IB: ERK
70
55
IB: AKT
70
55
IB: pAKT
55
IB: β-actin

Figure 2

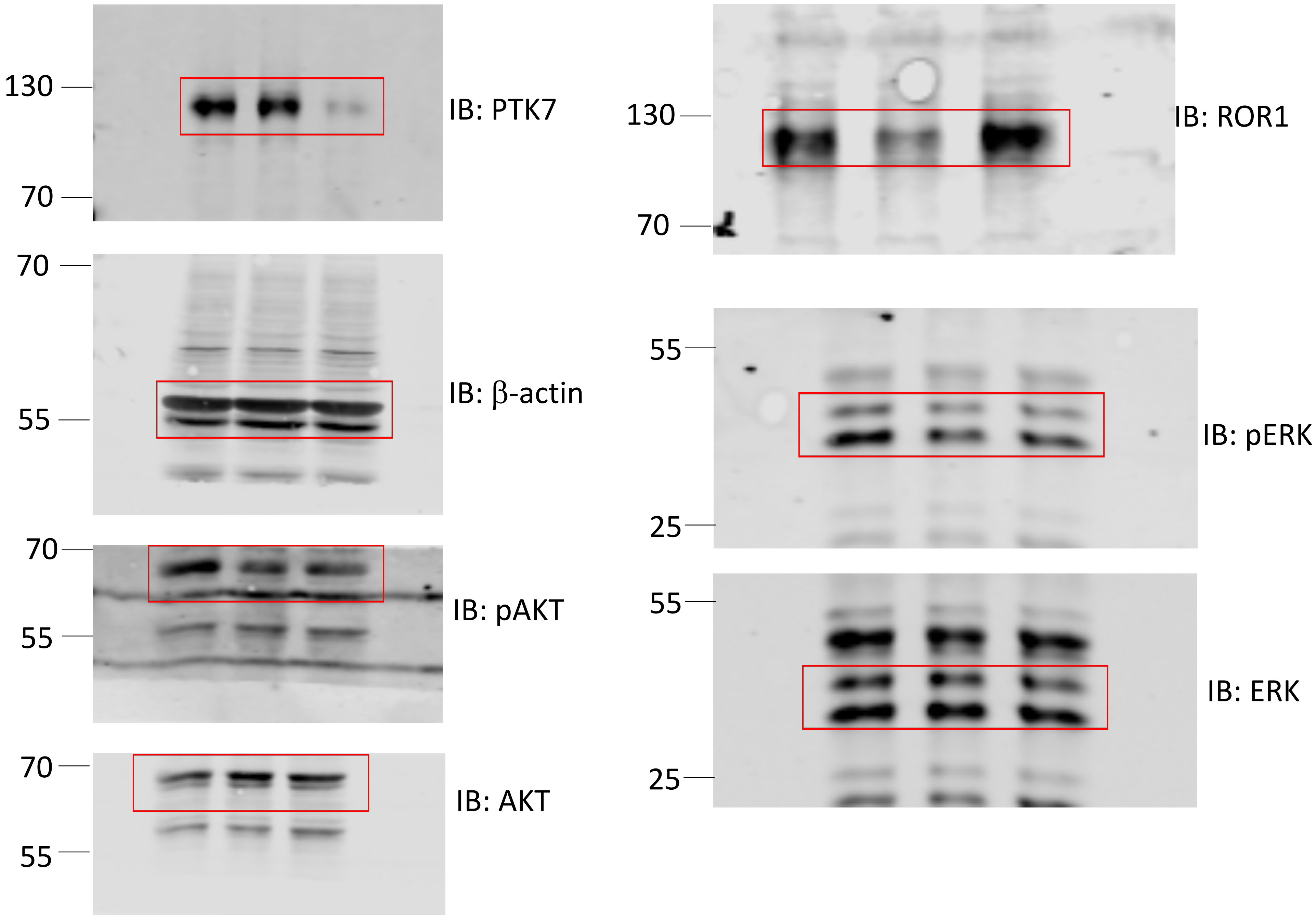

130
70
IB: PTK7
70
55
IB: β-actin
70
55
IB: pAKT
70
55
IB: AKT
130
70
IB: ROR1
55
25
IB: pERK
55
25
IB: ERK

Figure 2

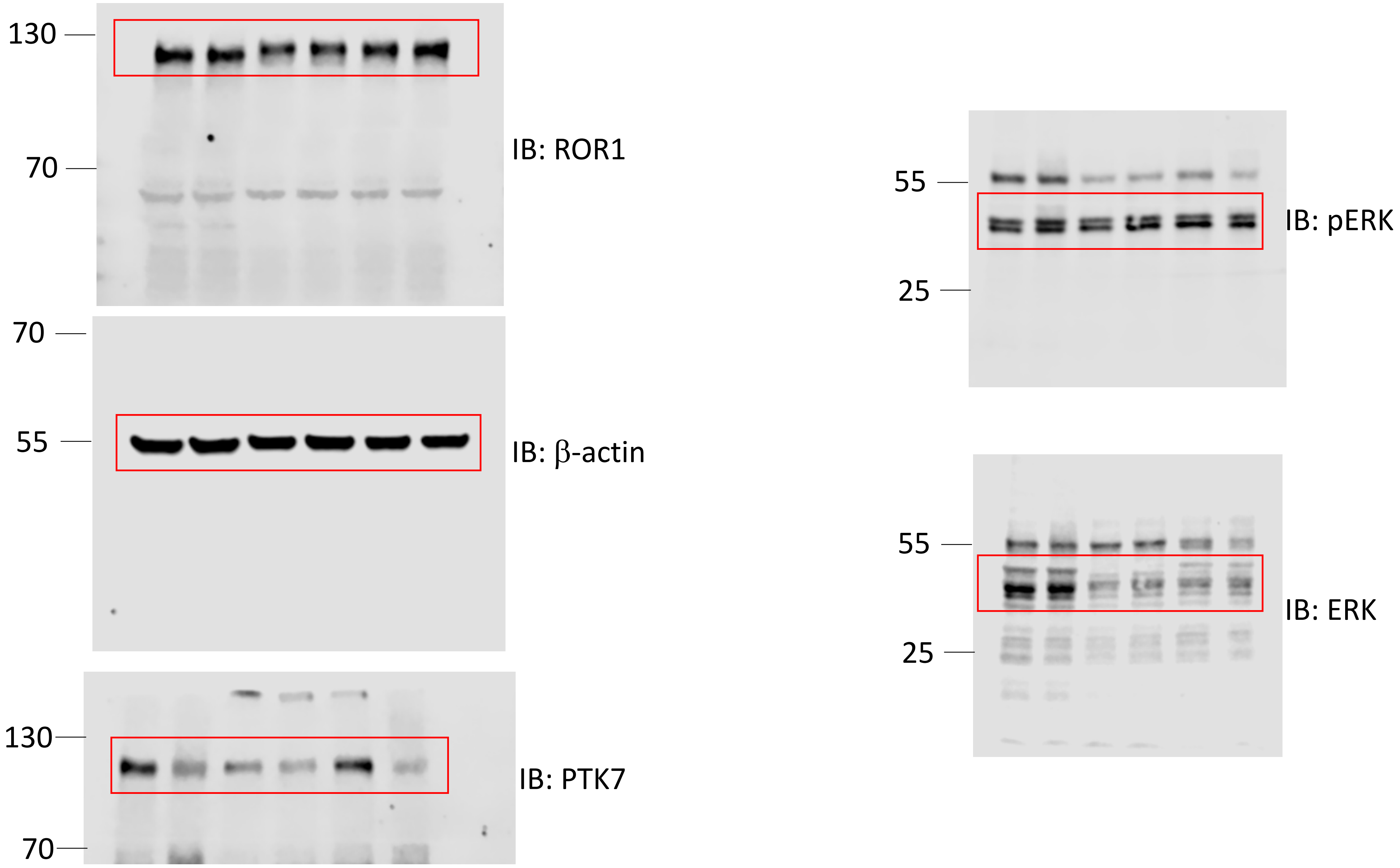

130
70
IB: ROR1
70
55
IB: β-actin
130
70
IB: PTK7
55
25
IB: pERK
55
25
IB: ERK

Figure 3

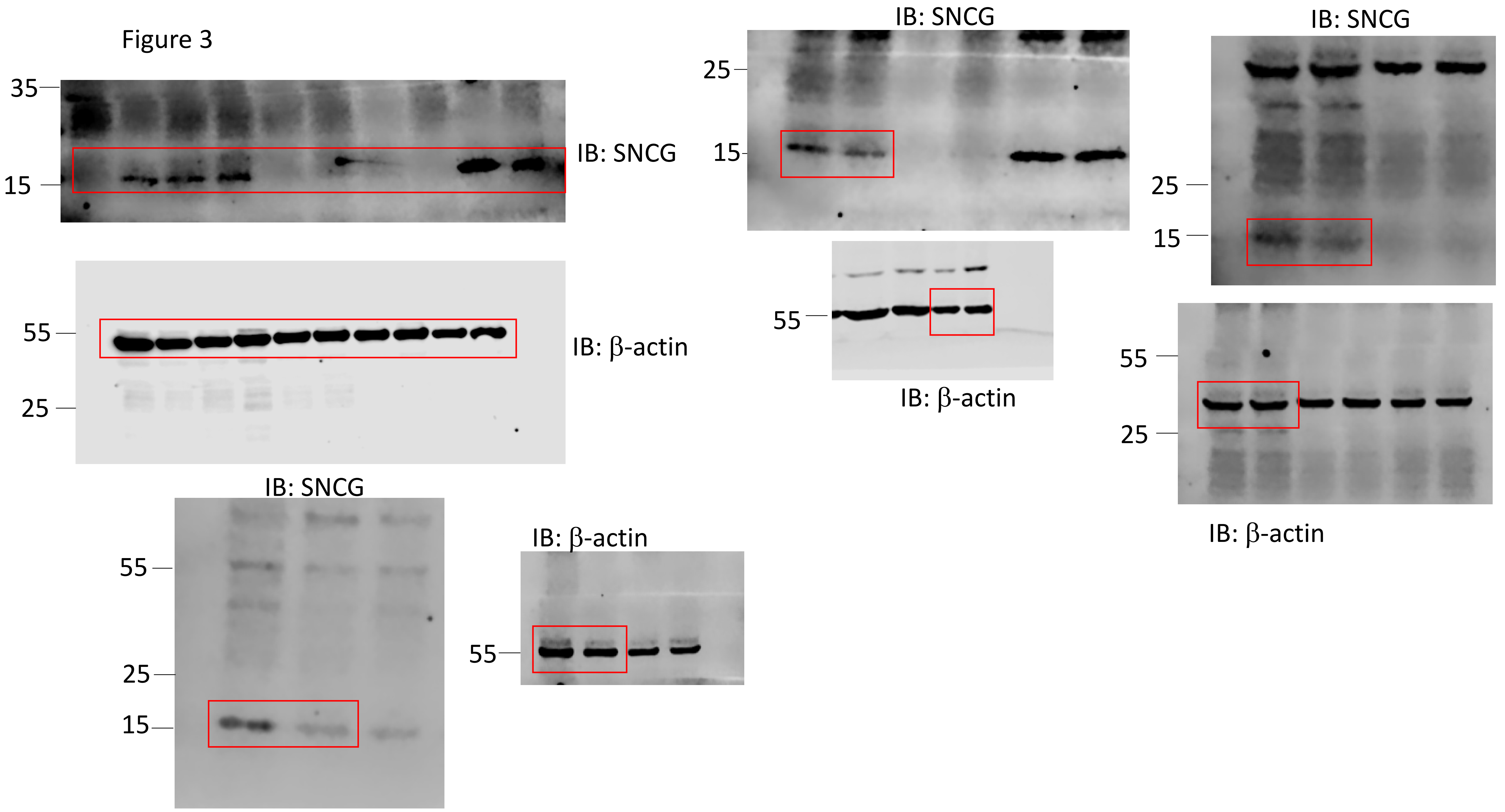

IB: SNCG
35
15
IB: β-actin
55
25
IB: SNCG
25
15
IB: β-actin
55
IB: SNCG
25
15
IB: β-actin
55
25
IB: SNCG
55
25
15
IB: β-actin
55

Figure 3

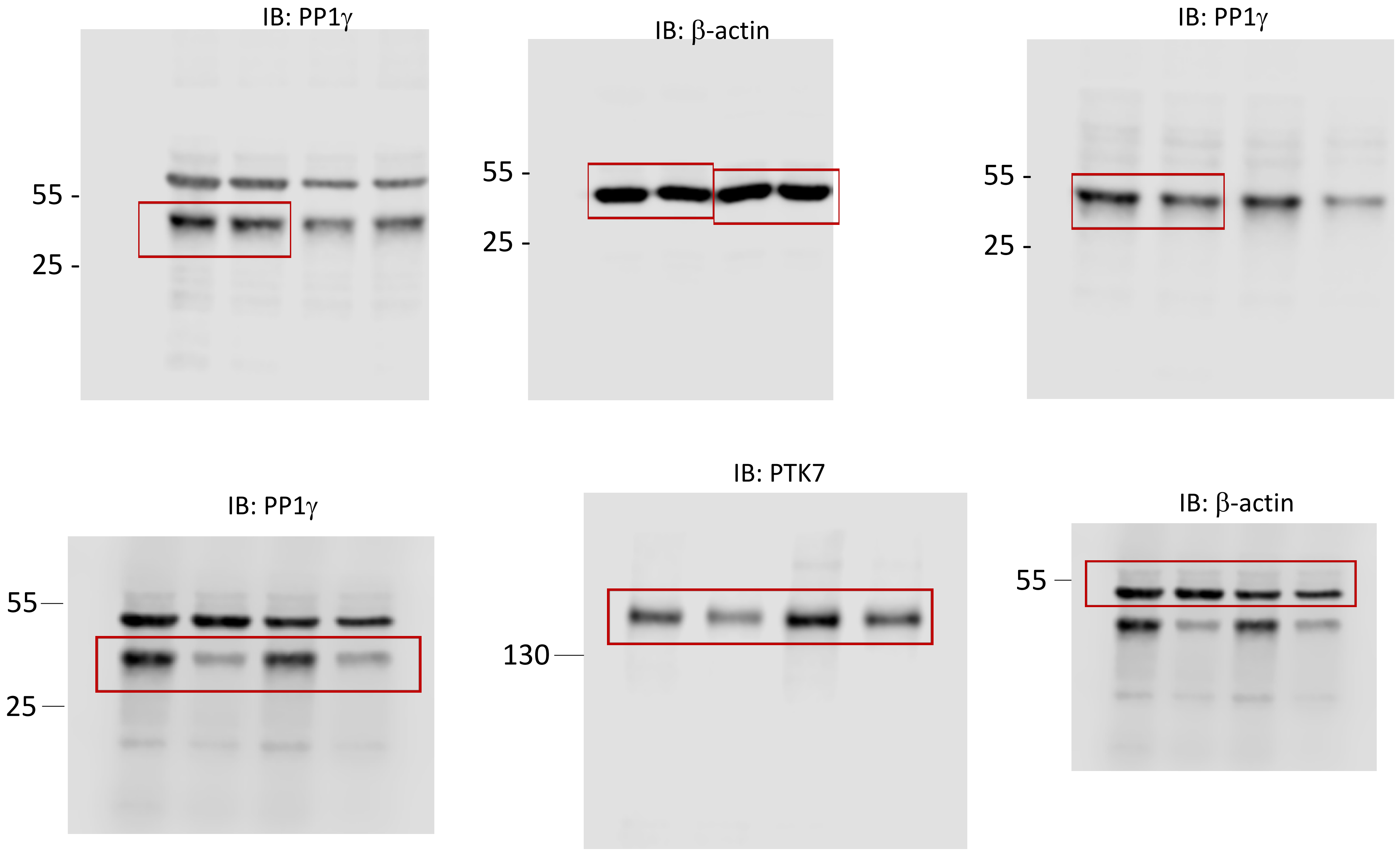
IB: PP1γ
55 -
25 -
IB: β-actin
55 -
25 -
IB: PP1γ
55 -
25 -
IB: PP1γ
55
25
IB: PTK7
130
IB: β-actin
55

Figure 3

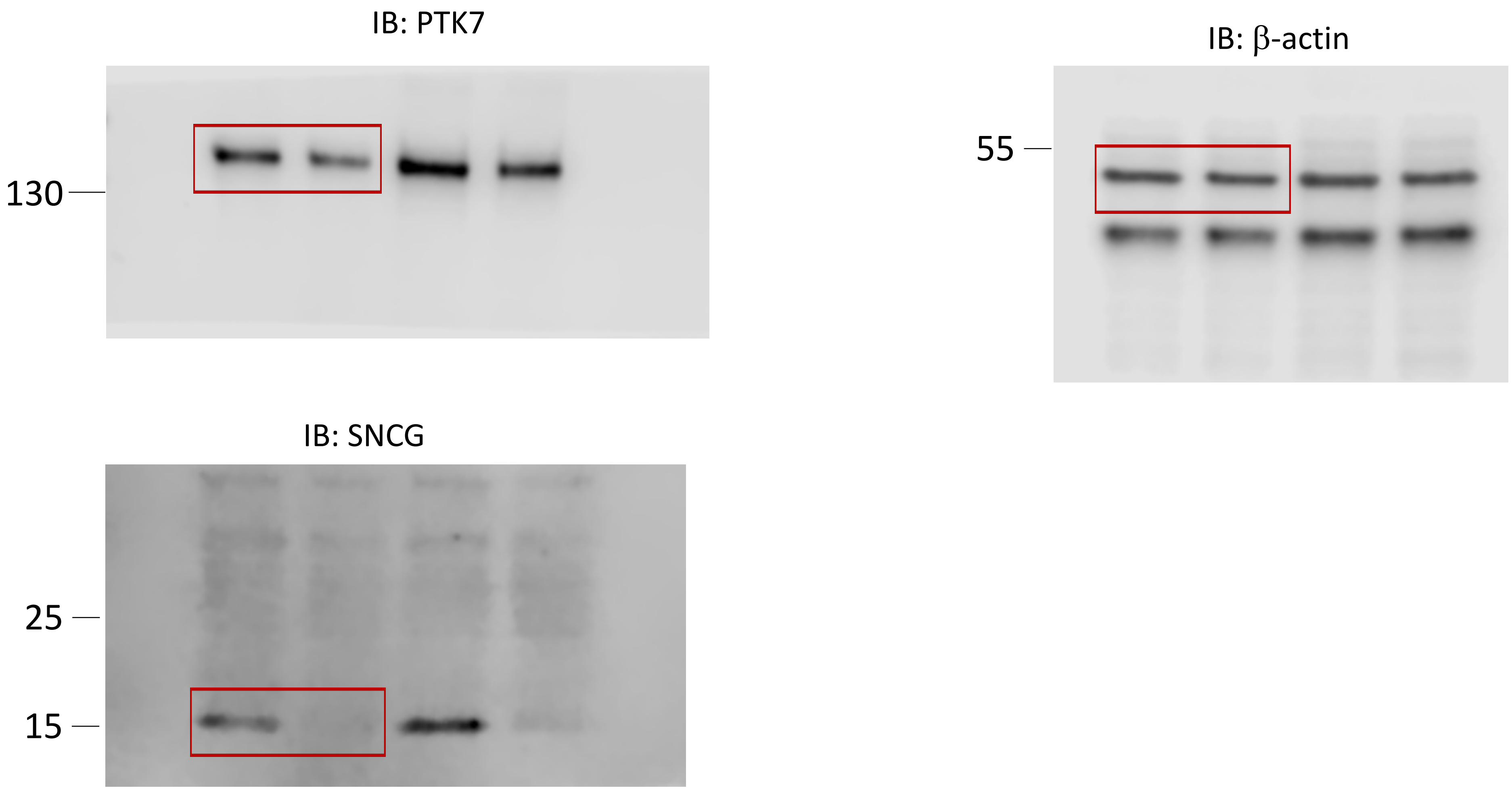

Figure 5

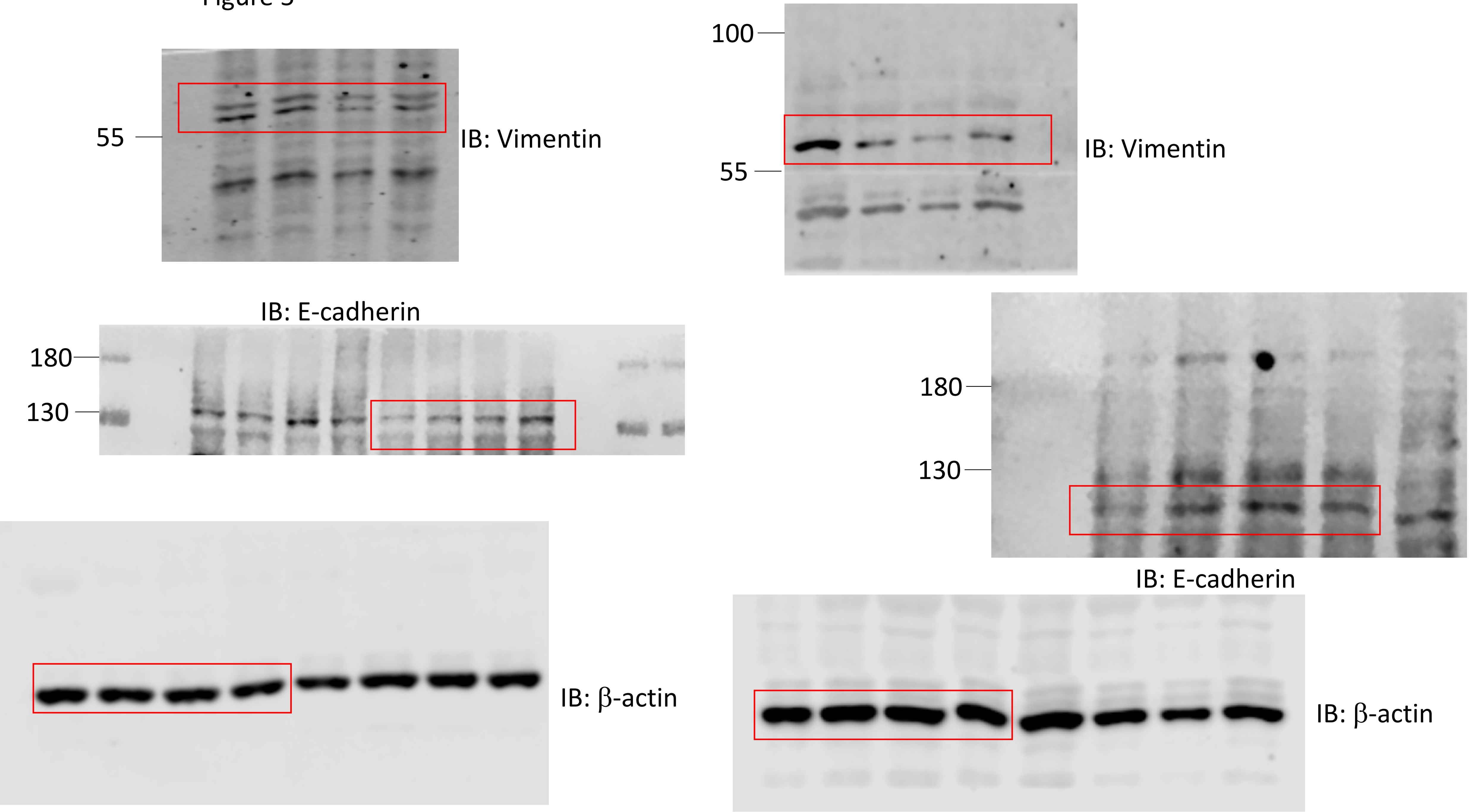
55
IB: Vimentin
100
55
IB: Vimentin
IB: E-cadherin
180
130
180
130
IB: E-cadherin
IB: β-actin
IB: β-actin
